# Supplementary material for: A nutrient responsive lipase mediates gut-brain communication to regulate insulin secretion in Drosophila
Source: Nat Commun. 2024 May 23;15:4410. doi: 10.1038/s41467-024-48851-8 (PMC11116528; doi:10.1038/s41467-024-48851-8)
Supplement: Supplementary file 1 — Supplementary Information [file 41467_2024_48851_MOESM1_ESM.pdf]

A nutrient responsive lipase mediates gut-brain communication to regulate insulin secretion in *Drosophila*

Alka Singh[1+], Kandahalli Venkataranganayaka Abhilasha [2+], Kathya R. Acharya[1,2,4], Haibo Liu[1], Niraj K. Nirala[3], Velayoudame Parthibane[2], Govind Kunduri[2], Thiruvaimozhi Abimannan[2], Jacob Tantalla[2], Lihua Julie Zhu[1], Jairaj K. Acharya[2*] and Usha R. Acharya[2*]

*Corresponding authors: Usha R. Acharya acharyaur@nih.gov

Jairaj K. Acharya acharyaj@mail.nih.gov

+ Alka Singh, Kandahalli Venkataranganayaka Abhilasha These authors contributed equally.

This file includes Supplementary Figures 1-16, Supplementary Table 1, Supplementary Table 2, Supplementary Legends and Supplementary References.

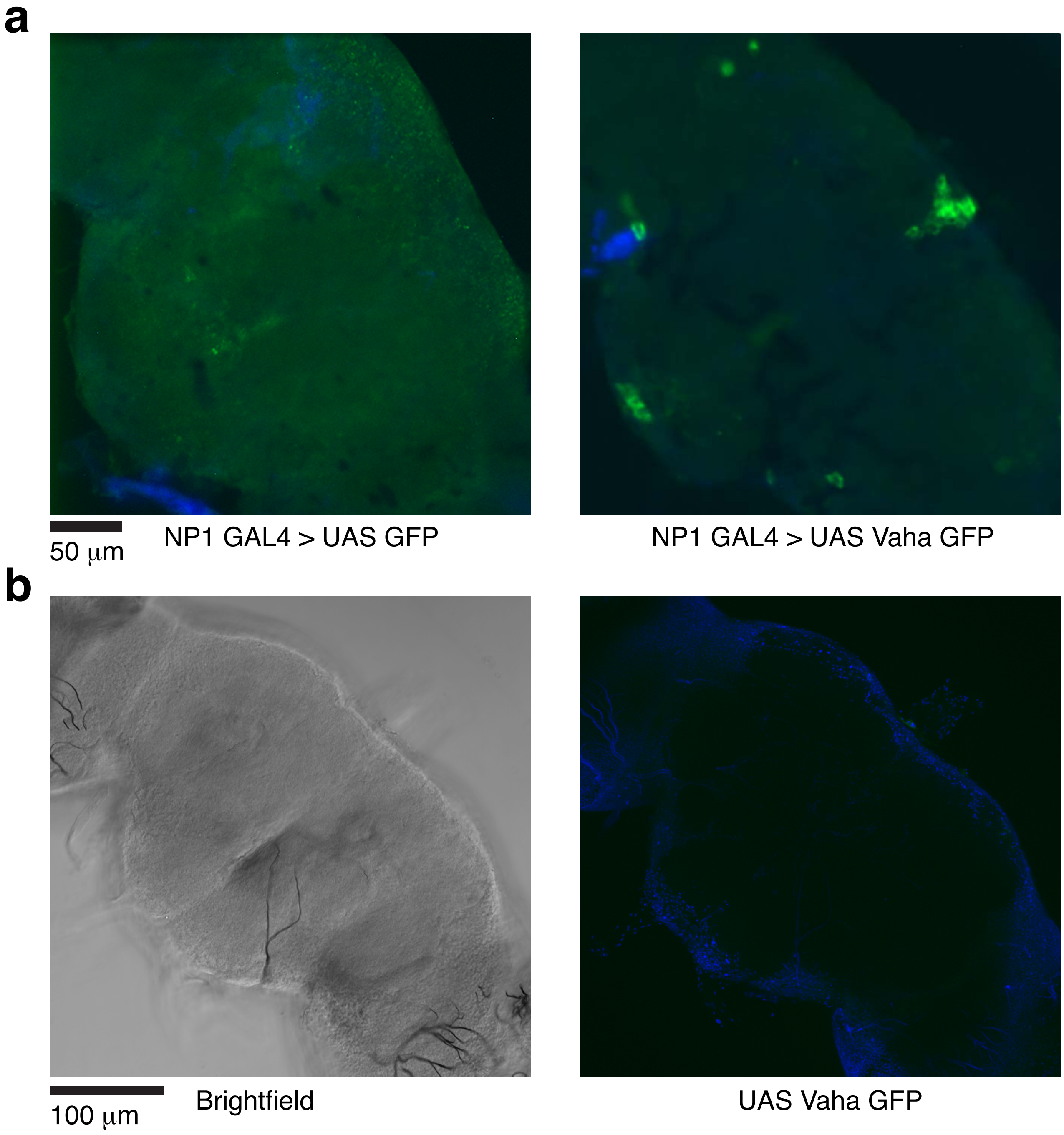

Supplementary Figure 1a, b

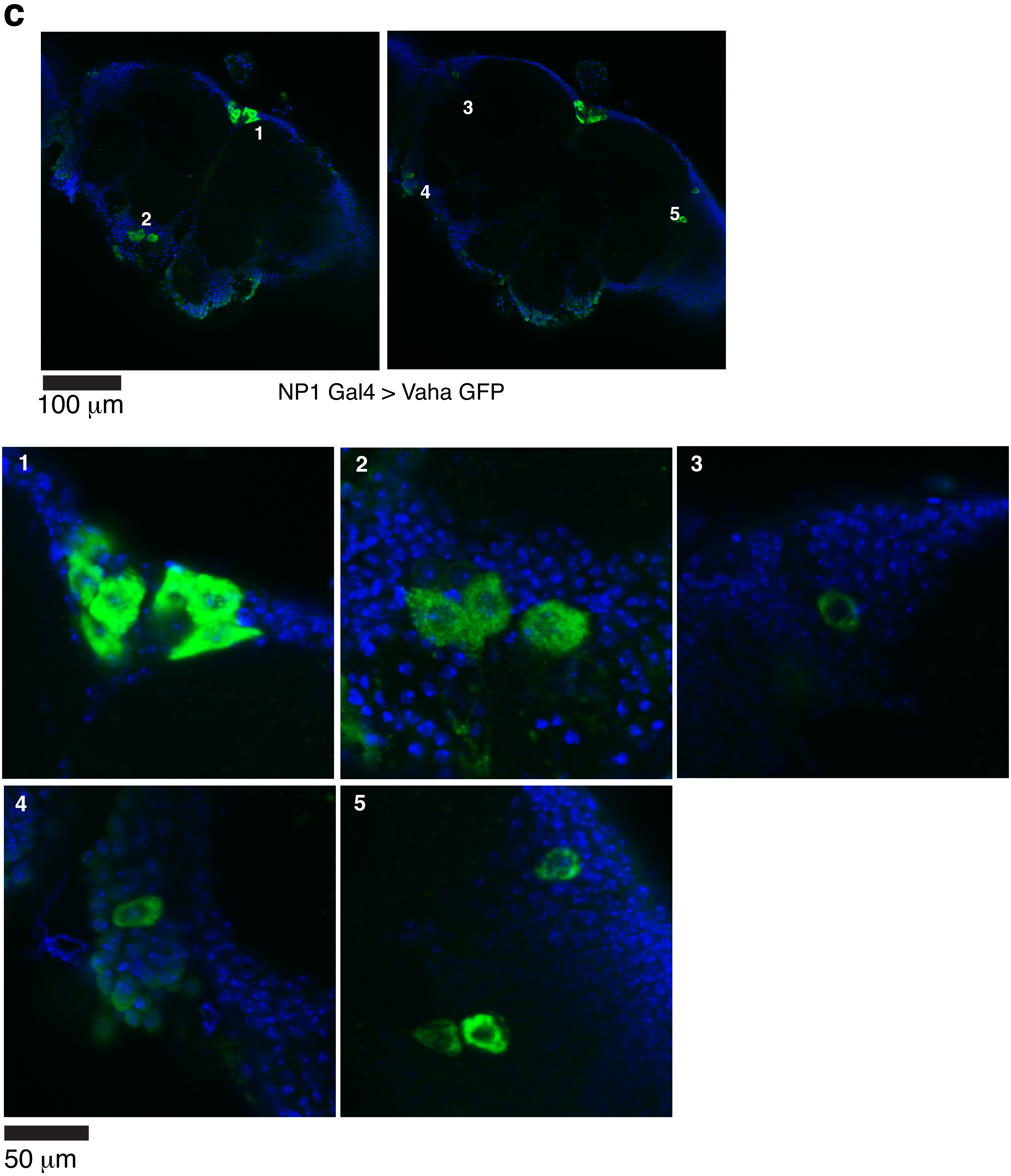

Supplementary
Figure 1c

Supplementary Fig. 1: Vaha expressed in the gut is detected in the PI region of the brain

a Immunostaining for GFP in brains dissected from 5-7day old adult flies expressing either UAS GFP or UAS Vaha GFP in the gut using NP1Gal4 driver. Nuclei are stained with DAPI (blue). The confocal images are projection of z stacks, and the images are representative of 6 brains, scale bar 50 μm.

b Immunostaining for GFP in brains dissected from 5-7day old adult UAS Vaha GFP transgenic flies without driver, nuclei are stained with DAPI (blue). The confocal images are projection of z stacks, and the images are representative of 6 brains, scale bar 100 μm.

c Immunostaining for GFP in brains dissected from 5-7day old adult flies expressing UAS Vaha GFP in the gut using NP1Gal4 driver. Nuclei are stained with DAPI (blue). GFP staining is observed in the pars intercerebralis region of the brain in Vaha GFP expressing flies (1), the subesophageal ganglion area (2) and additional areas marked 3, 4, 5. The confocal images are z stacks, and the images are representative of 6 brains, scale bar 100 μm. High magnification views of areas 1-5 are shown, scale bar 50 μm.

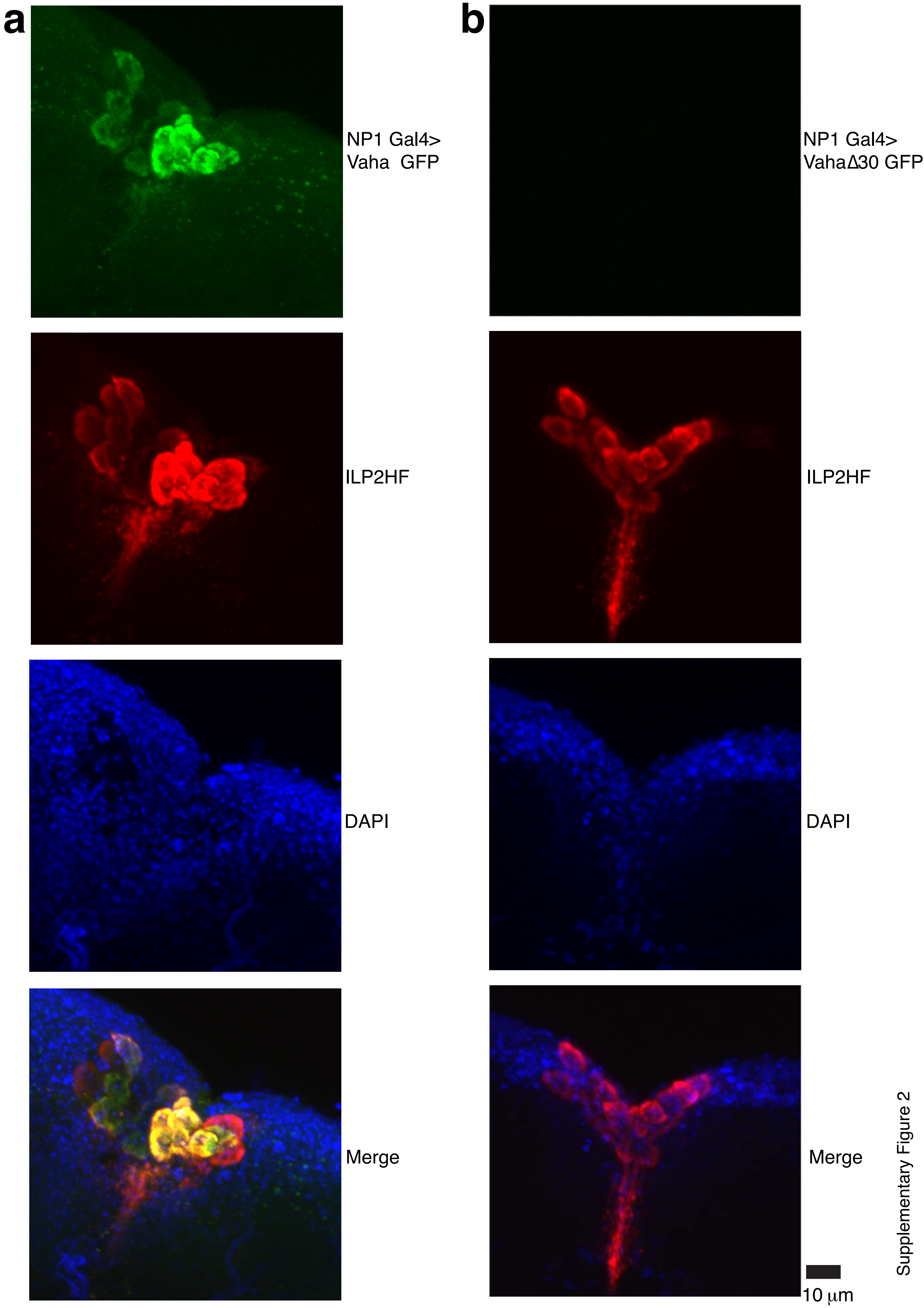

Supplementary Figure 2

Supplementary Fig. 2: Vaha expressed in the gut is enriched in the IPCs

a The panels show immunostaining of the IPC region of the brain (from main Fig. 1c) expressing Vaha GFP (anti GFP, green) and ILP2HF (anti HA, red). The genotype of the flies is *w*⁻; NP1Gal4/+; UASVaha GFP/gd2HF. ILP2HF (gd2HF) marks the insulin-producing cells. Nuclei are stained with DAPI (blue). Merged image shows overlap of GFP and HA staining in a majority of the IPCs. The confocal images are projection of z stacks, and the images are representative of 6 brains, scale bar 10 µm.

b The panels show immunostaining of the IPC region of the brain (from main Fig. 1d) expressing Vaha Δ30GFP in the gut and ILP2HF under the control of its own promoter. Nuclei are stained with DAPI (blue). The genotype of the flies is *w*⁻; NP1Gal4/+; Vaha Δ30GFP /gd2HF. GFP expression is not detected in the IPCs. The confocal images are projection of z stacks, and the images are representative of 6 brains, scale bar 10 µm.

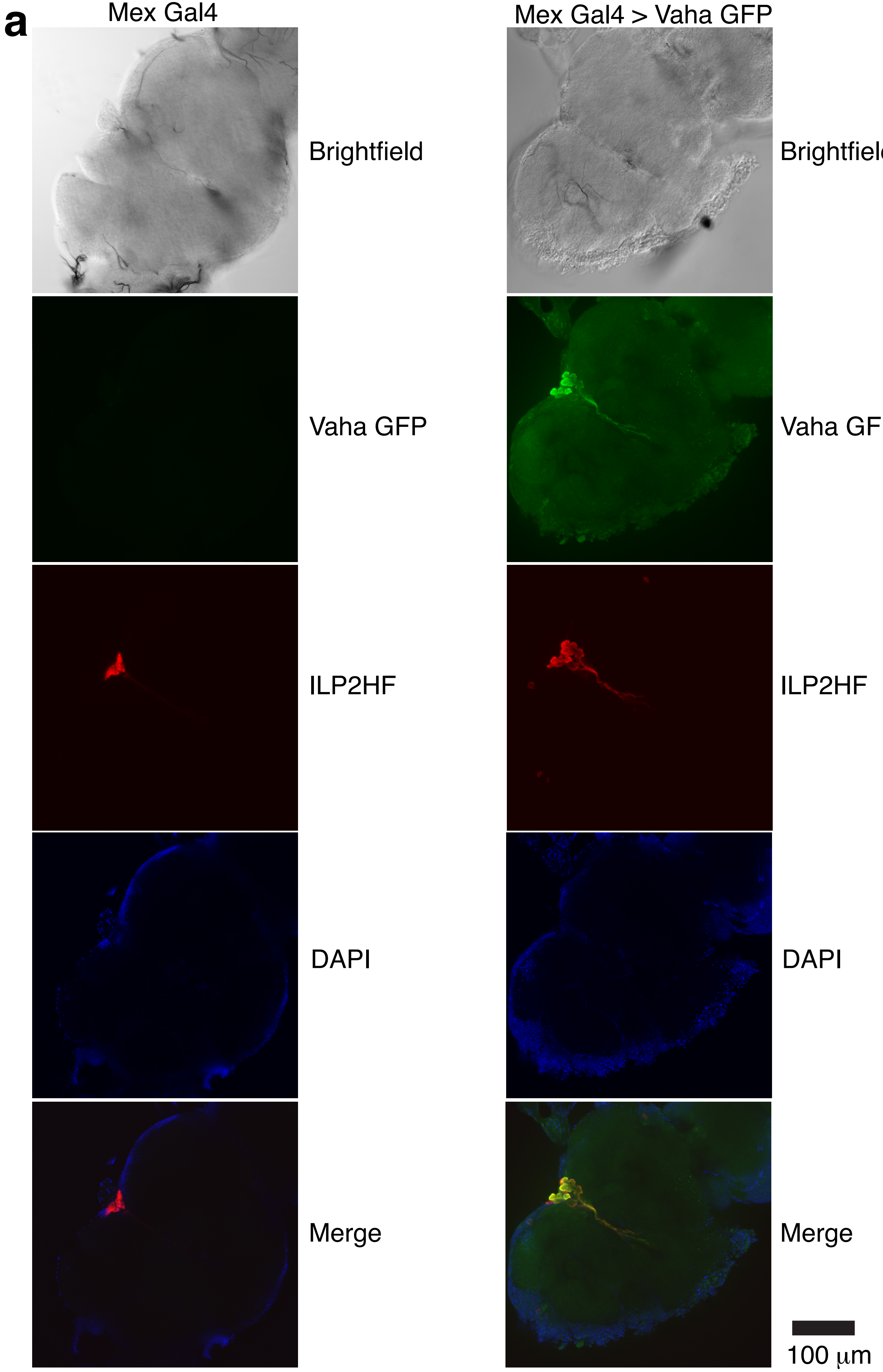

Supplementary Figure 3a

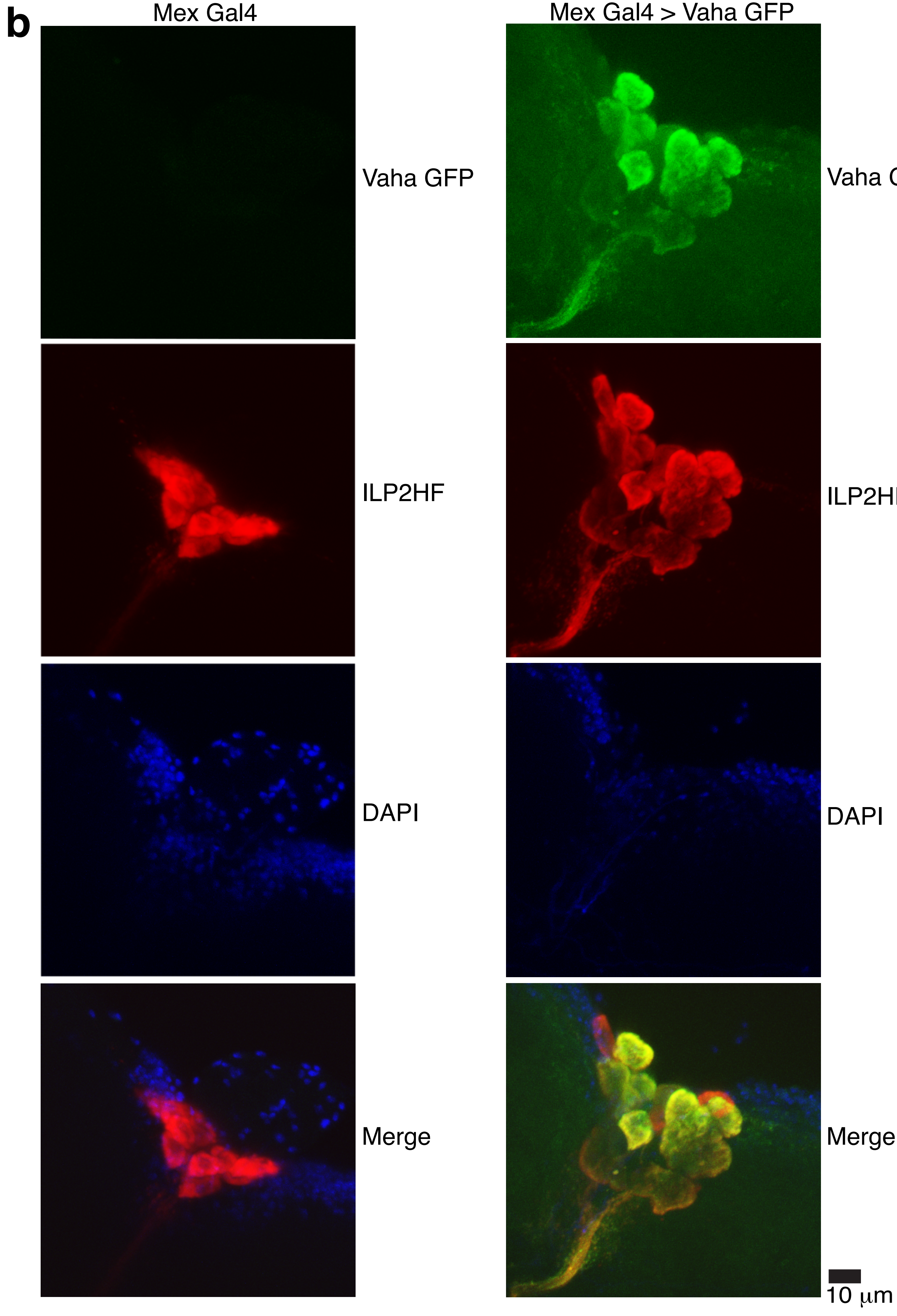

Supplementary Figure 3b

Supplementary Fig. 3: Vaha expressed using a second gut Gal4 driver is enriched in the IPCs

a Immunostaining of Vaha GFP (anti GFP, green) and ILP2HF (anti HA, red) in brain dissected from 5-7day old adult flies expressing Vaha GFP in the gut using Mex1 Gal4 driver and ILP2HF under the control of its own promoter. Nuclei are stained with DAPI (blue). Merged image shows overlap of GFP and HA staining in the IPCs. The confocal images are projection of z stacks, and the images are representative of 6 brains, scale bar 100 μm.

b The panels show magnified views of the IPC region from the above brains. Merged image shows overlap of GFP and HA staining in the IPCs when Vaha GFP is driven using Mex1 Gal4 driver. The confocal images are projection of z stacks, and the images are representative of 6 brains, scale bar 10 μm.

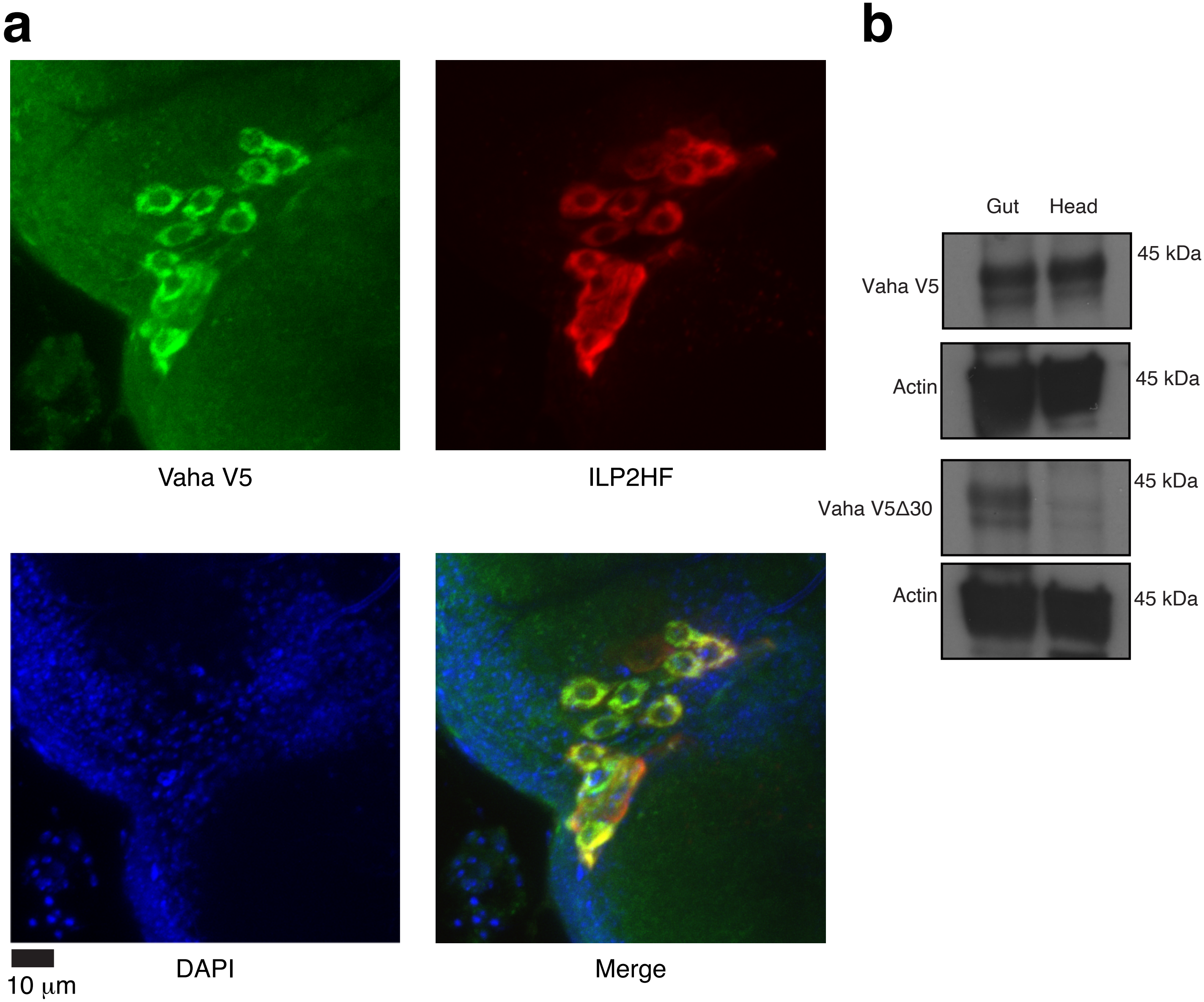

Supplementary Figure 4

Supplementary Fig. 4: Vaha expressed using its own promoter is enriched in the IPCs

a Immunostaining for Vaha V5 (anti V5, green) and ILP2HF (anti HA, red) in the IPC region of brain shown in main Fig.1e from 5-7day old adult flies expressing Vaha V5 under the control of its own promoter and ILP2HF to mark the IPCs (*w*-; Vaha V5/VahaV5; gd2HF/gd2HF). Nuclei are stained with DAPI (blue). Merged image shows overlap of V5 and HA staining in a majority of the IPCs. The confocal images are projection of z stacks, and images are representative of 6 brains, scale bar 10 μm.

b Immunoblotting with V5 antibody of extracts prepared from 4 dissected guts and 4 dissected heads from flies expressing Vaha V5 under the control of its own promoter (top panel). Immunoblotting with V5 antibody of extracts prepared from 4 dissected guts and 4 dissected heads from flies expressing the signal peptide deleted construct Vaha Δ30V5 under the control of its own promoter (bottom panel). Vaha V5 is detected in the gut and head extracts in the top panel while it is detected only in the gut in the signal peptide lacking extracts in the bottom panel. Actin is used as a loading control. The blot is a representative image of 3 independent experiments. Source data are provided as a source data file.

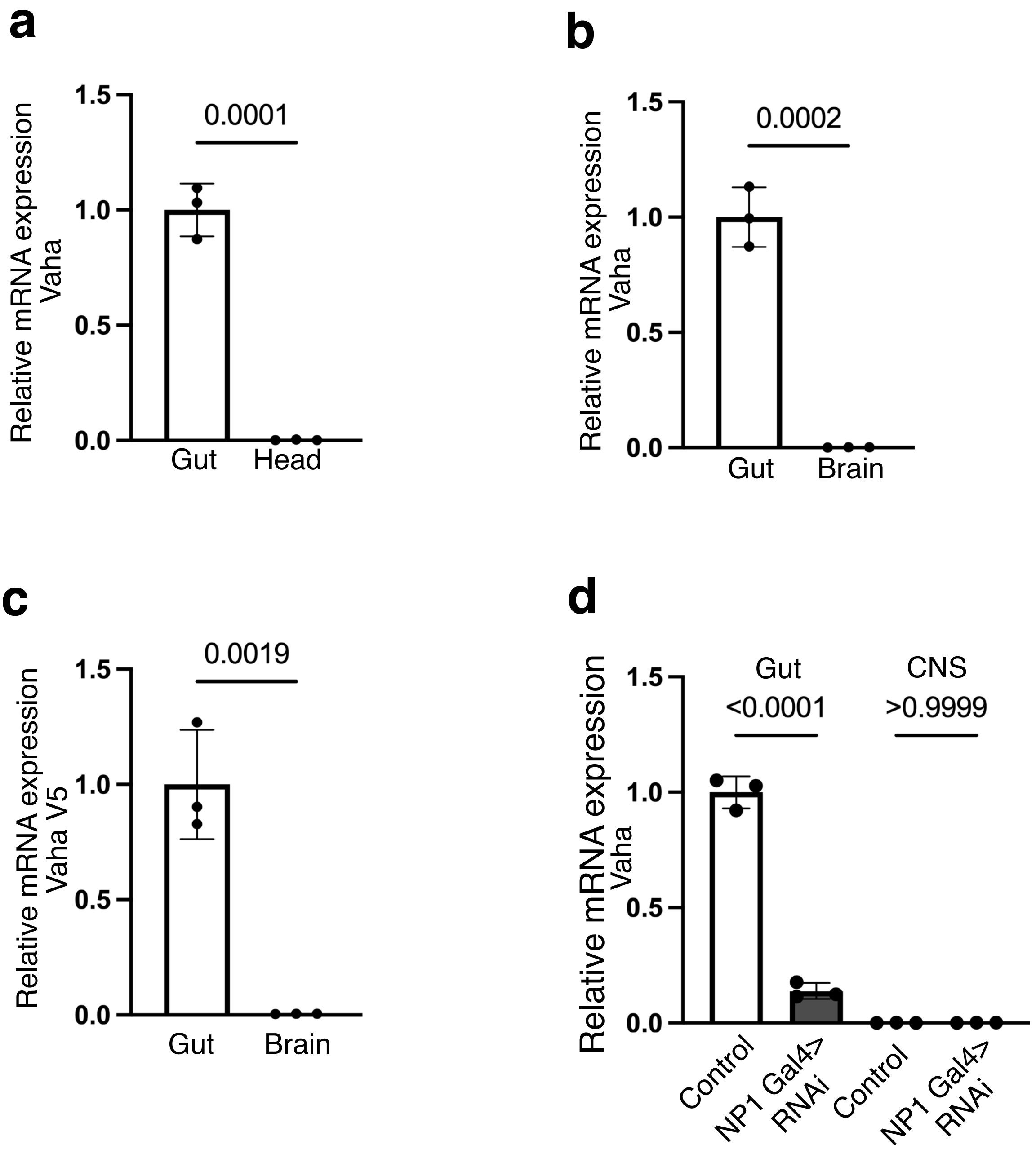

Supplementary Figure 5

Supplementary Fig. 5: Vaha is synthesized in the gut

a RT-qPCR analysis of Vaha mRNA level in gut and head samples isolated from w^{1118}. *Vaha* expression is normalized to *GAPDH*. n=3, 30 guts and 40 heads per replicate, data is assessed by two-tailed Student's t-test and presented as mean ± SD. Source data are provided as a source data file.

b RT-qPCR analysis of Vaha mRNA level in gut and brain samples isolated from w^{1118}. *Vaha* expression is normalized to *GAPDH*. n=3, 10 guts and 10 brains per replicate, data is assessed by two-tailed Student's t-test and presented as mean ± SD. Source data are provided as a source data file.

c RT-qPCR analysis of Vaha V5 mRNA level in gut and brain samples isolated from Vaha V5 flies. *Vaha V5* expression is normalized to *GAPDH*. n=3, 10 guts and 10 brains per replicate, data is assessed by two-tailed Student's t-test and presented as mean ± SD. Source data are provided as a source data file.

d RT-qPCR analysis of Vaha mRNA level in gut and CNS samples isolated from control and gut specific Vaha RNAi flies. *Vaha* expression is normalized to *GAPDH*. n=3, 10 guts and 10 CNS per replicate, data is assessed by two-tailed Student's t-test and presented as mean ± SD. Source data are provided as a source data file.

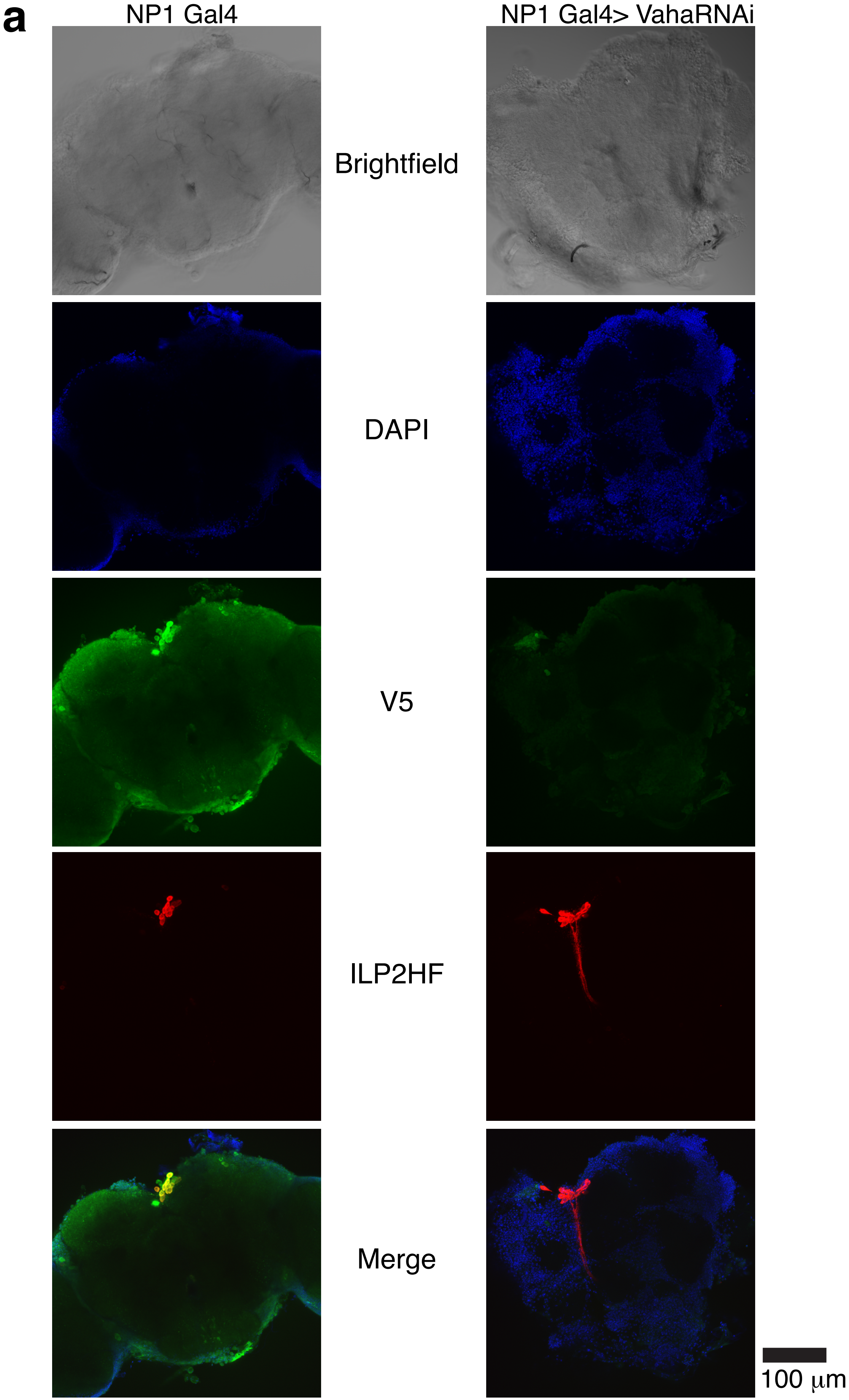

Supplementary Figure 6a

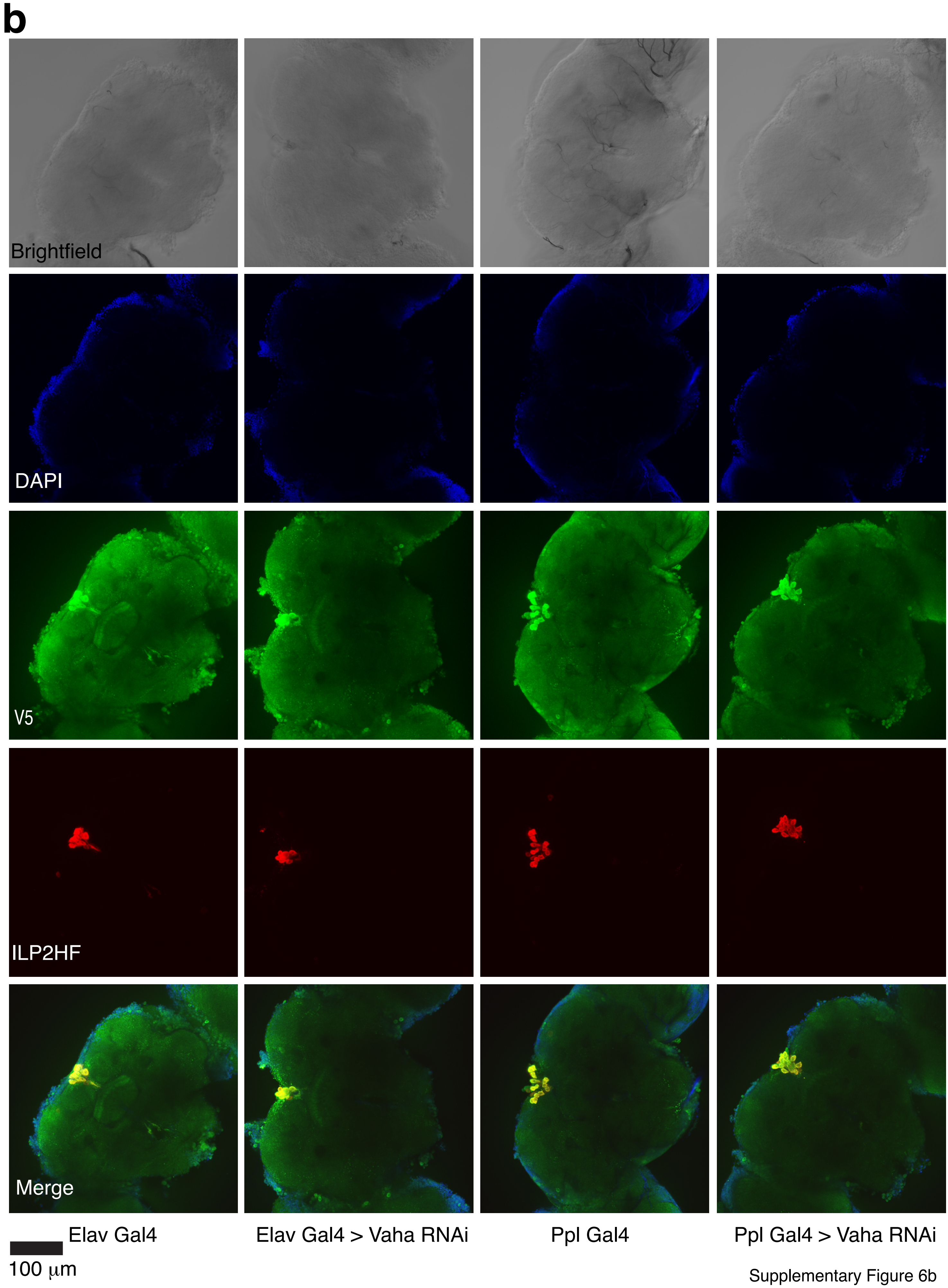

Supplementary Figure 6b

Supplementary Fig. 6: Gut specific knockdown of Vaha significantly reduces Vaha V5 staining in the IPCs

a Vaha RNAi in the gut (w^-; NP1 Gal4/Vaha V5; Vaha RNAi/gd2HF) using NP1 Gal4 driver and imaging of Vaha V5 in the IPCs. Control flies are w^-; NP1 Gal4/Vaha V5; +/gd2HF. IPCs are marked by ILP2HF, and nuclei are stained with DAPI. The confocal images are projection of z stacks, and the images are representative of 6 brains, scale bar 100 μm.

b Vaha RNAi in the neurons (w^-; Elav Gal4/Vaha V5; Vaha RNAi/gd2HF) and fat body (w^-; Ppl Gal4/Vaha V5; Vaha RNAi/gd2HF) using specific Gal4 drivers and imaging of Vaha V5 in the IPCs. Control flies are neurons (w^-; Elav Gal4/Vaha V5; +/gd2HF) and fat body (w^-; Ppl Gal4/Vaha V5; +/gd2HF). IPCs are marked by ILP2HF, and nuclei are stained with DAPI. The confocal images are projection of z stacks, and the images are representative of 6 brains, scale bar 100 μm.

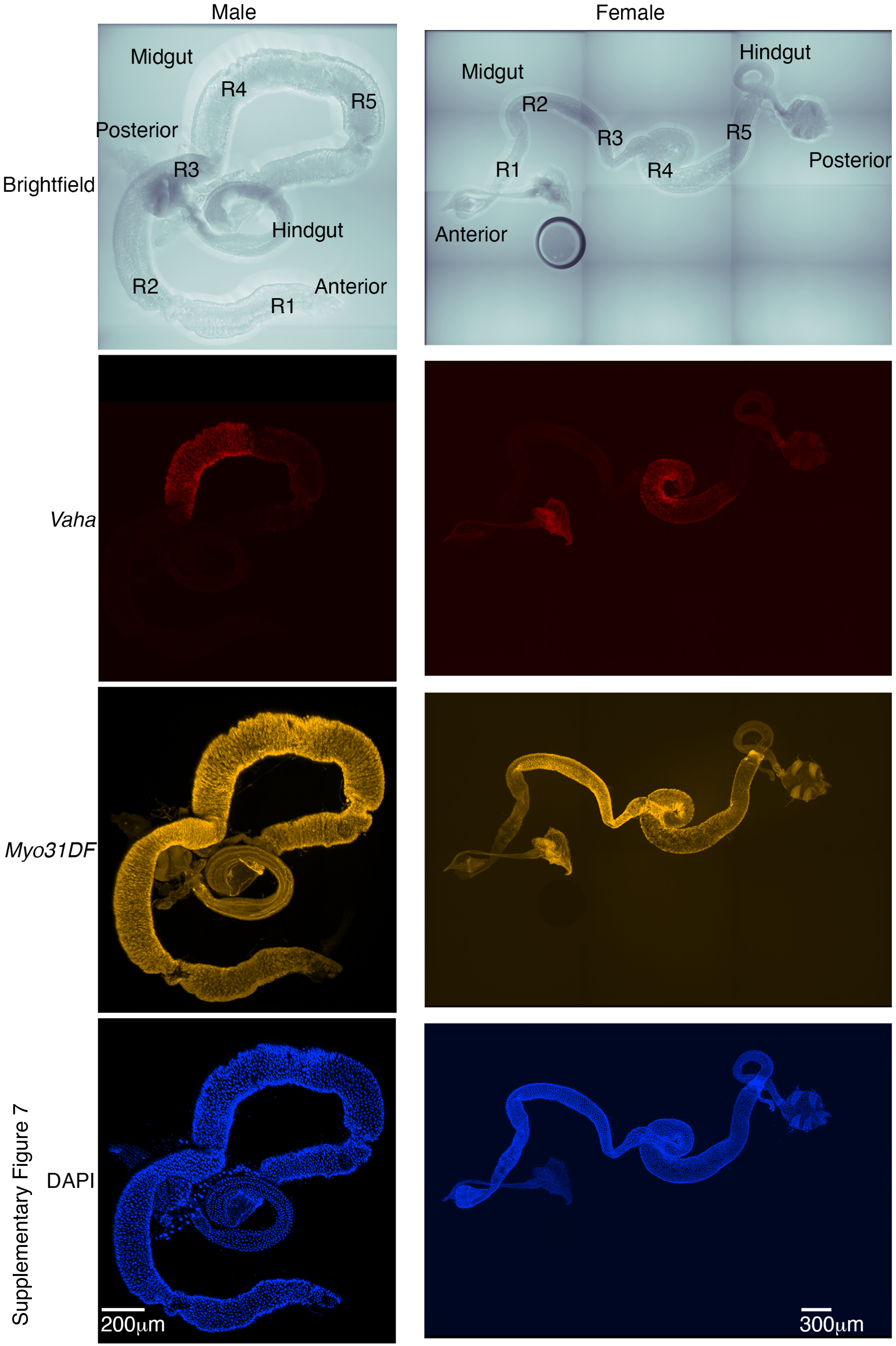

Supplementary Figure 7

Supplementary Fig. 7: Hybridization chain reaction to visualize Vaha transcript in w^{1118} gut.

The left panel shows male gut while the right panel shows female gut. Brightfield images depict the midgut with the R1-R5 subdivisions. *Vaha* staining is in red, *myo31DF* staining is pseudocolored orange and nuclei are stained with DAPI (blue). *Vaha* transcript can be visualized in the R4 region of the midgut. The confocal images are z projections, and images are representative of 3 guts, scale bar 200 µm (left panel), 300 µm (right panel).

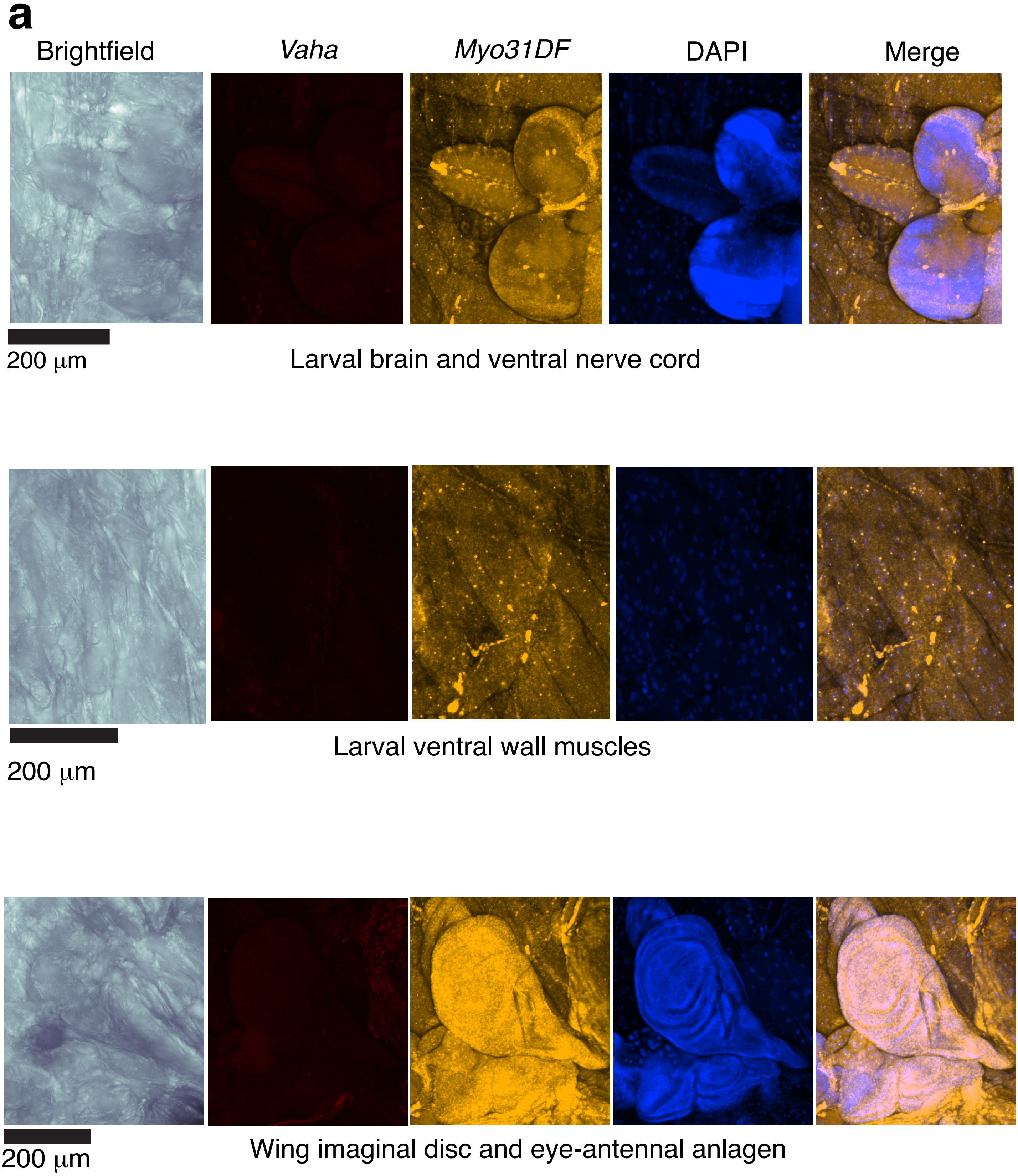

Supplementary Figure 8a

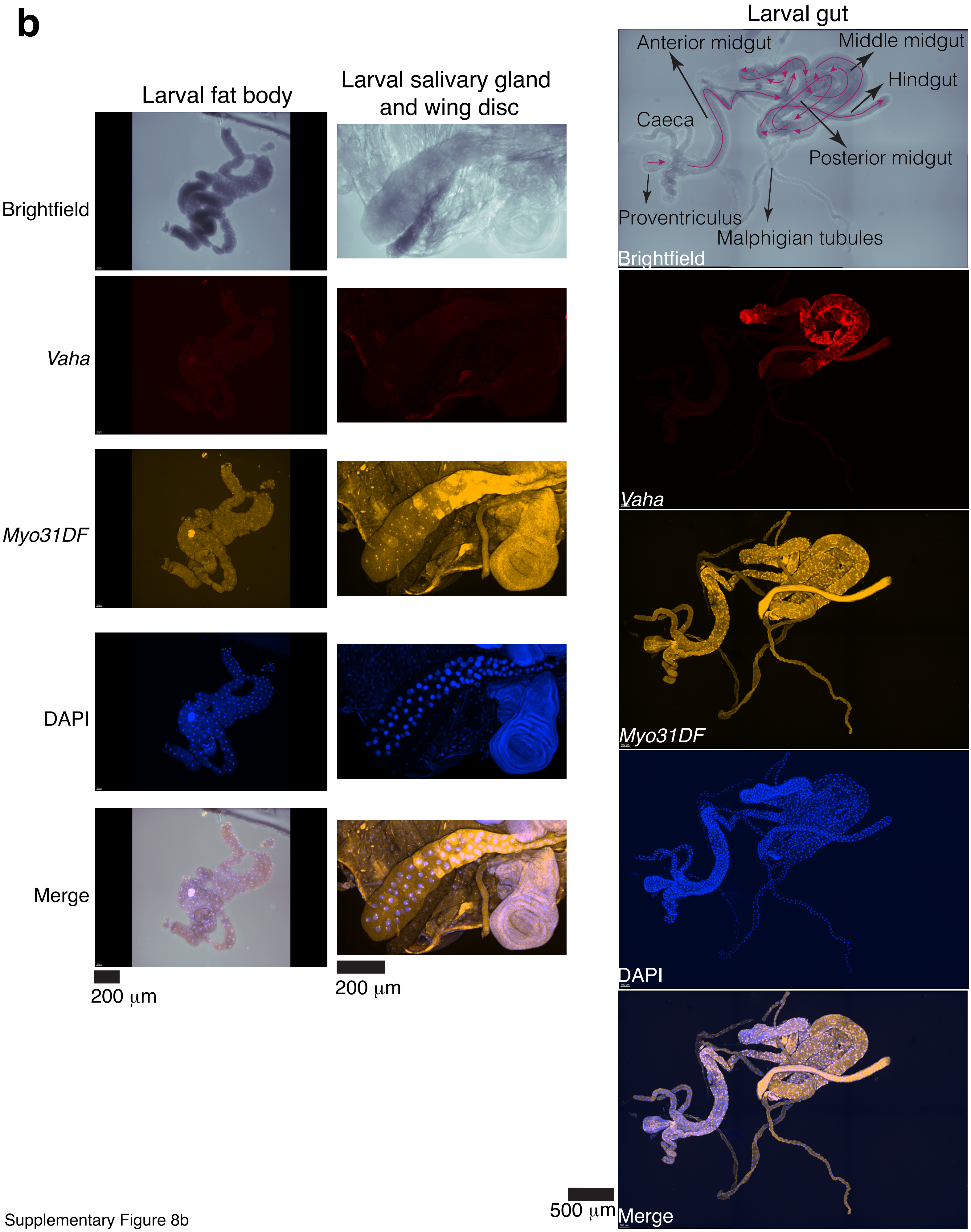

Supplementary Figure 8b

Supplementary Fig. 8: Hybridization chain reaction to visualize *Vaha* transcript in w^{1118} third instar larvae.

a Third instar larvae are dissected, and HCR is carried out on different organs. *Vaha* staining is in red, *myo31DF* staining is pseudocolored orange and nuclei are stained with DAPI (blue). *Vaha* transcript cannot be visualized in the larval brain, ventral nerve cord, ventral wall muscles, wing and eye imaginal discs. The confocal images are z projections, and images are representative of 3 larvae, scale bar 200 μm.

b Third instar larvae are dissected, and HCR is carried out on different organs. *Vaha* staining is in red, *myo31DF* staining is pseudocolored orange and nuclei are stained with DAPI (blue). *Vaha* transcript can be visualized in the larval gut but not in the fat body or salivary gland. The confocal images are z projections, and images are representative of 3 larvae, scale bar 200 μm (first and second panels), 500 μm (third panel).

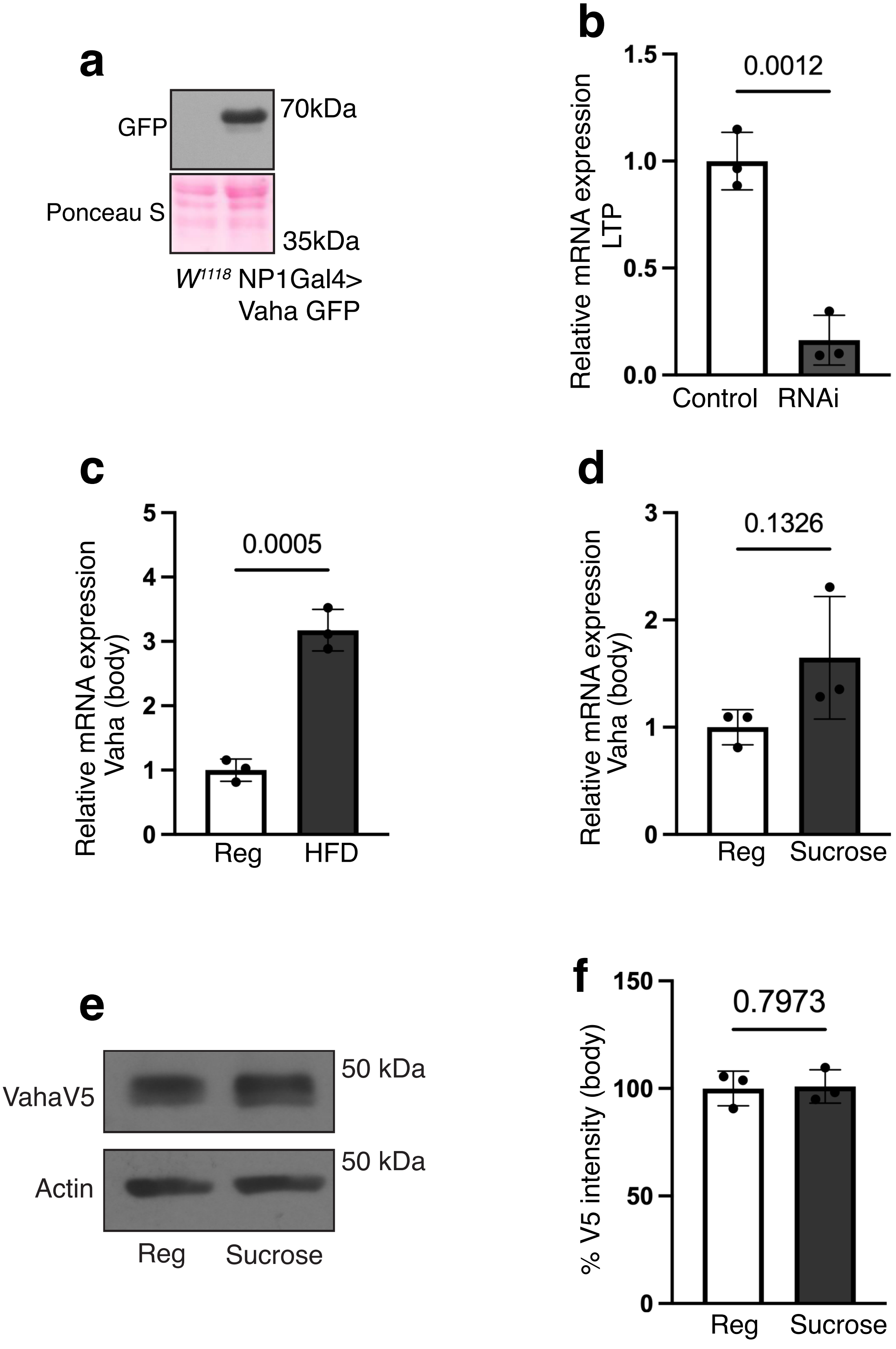

Supplementary Figure 9

Supplementary Fig. 9: Vaha expression is stimulated by fat

a Immunoblotting with GFP antibody of hemolymph collected from *w*$^{-}$; NP1 Gal4/+; UAS Vaha GFP/+ flies. Vaha GFP is detected in the hemolymph. n=3, hemolymph from 40 flies per replicate. Source data are provided as a source data file.

b RT-qPCR analysis of *LTP* mRNA level in body samples after LTP RNAi in the fat body. *LTP* expression is normalized to *GAPDH*. n=3, 10 flies per replicate, data is assessed by two-tailed Student's t-test and presented as mean ± SD. Source data are provided as a source data file.

c RT-qPCR analysis of *Vaha* mRNA level in body samples isolated from w^{1118} that were fasted overnight and put on regular food or high fat food for 1 h. *Vaha* expression is normalized to *GAPDH*. *Vaha* transcript level is higher on HFD compared to regular food. RNA was isolated from 30 flies for each replicate. n=3, data is assessed by two-tailed Student's t-test and presented as mean ± SD. Source data are provided as a source data file.

d RT-qPCR analysis of *Vaha* mRNA level in body samples isolated from w^{1118} that were fasted overnight and put on regular food or high sucrose food (2M sucrose) for 1 h. *Vaha* expression is normalized to *GAPDH*. RNA was isolated from 30 flies for each replicate. n=3, data is assessed by two-tailed Student's t-test and presented as mean ± SD. Source data are provided as a source data file.

e Immunoblotting for Vaha V5 protein expression in body extracts from flies raised on regular food (Reg) and high sucrose food (Sucrose) for 1 h after starvation overnight. One body is used per replicate. Actin is used as a loading control. Source data are provided as a source data file.

f Quantification of the blots shown in e. Vaha V5 expression is not different in sucrose containing food compared to regular food. n=3, data is assessed by two-tailed paired t-test and presented as mean ± SD. Source data are provided as a source data file.

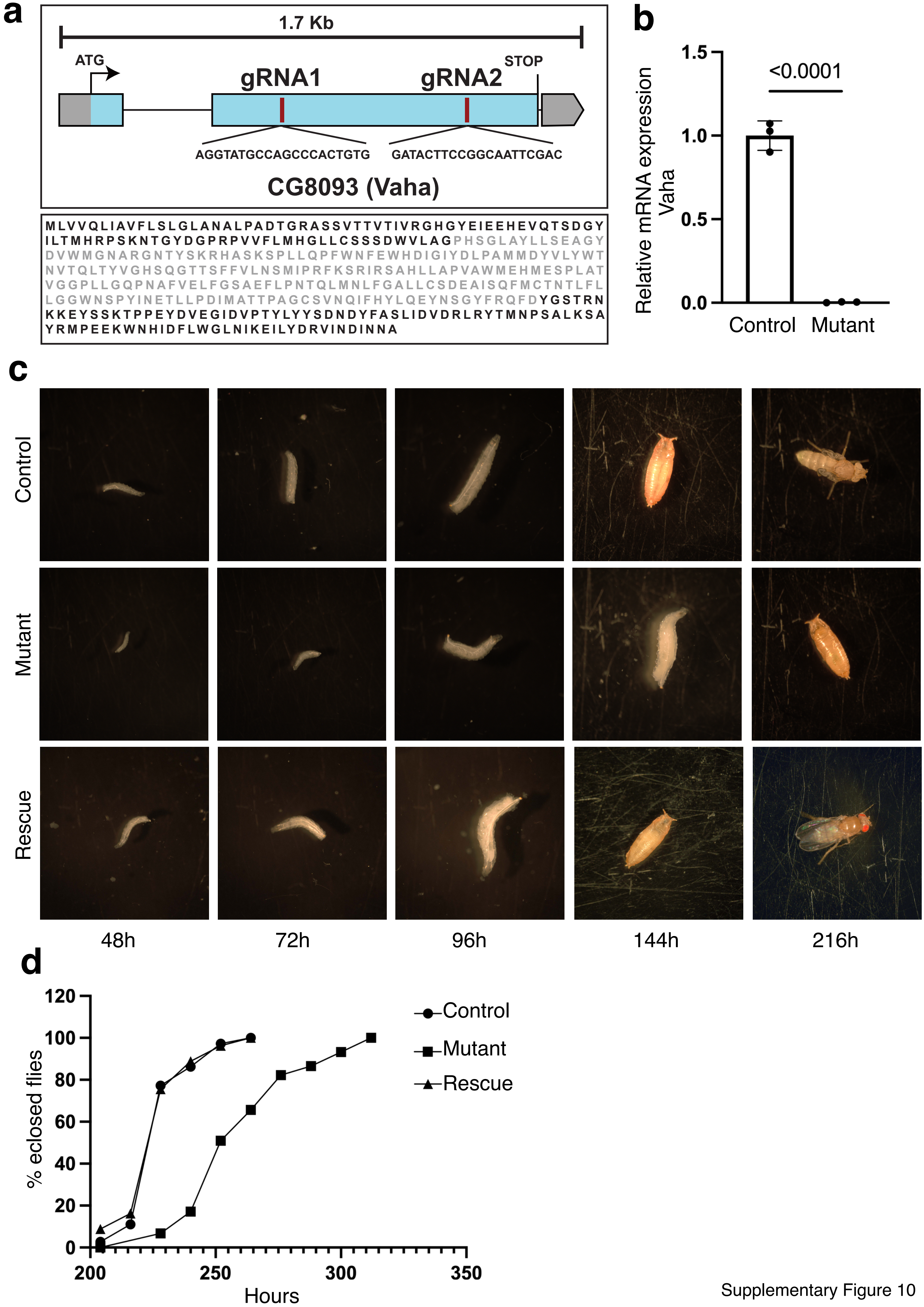

Supplementary Figure 10

Supplementary Fig. 10: Generation of *vaha* deletion mutant using CRISPR-Cas9 editing technology

a Outline of the CRISPR-Cas9 scheme used to generate *vaha* mutants. The two guide RNAs targeting the coding regions towards the N and C terminal parts are shown. The gRNAs are integrated into pCFD4 vector, the assembled plasmid is used to create gRNA transgenic lines which are crossed to nos-cas9 flies to induce a germline knockout. The knockout is sequenced and the region that would be deleted in the protein is shown in grey.

b RT-qPCR analysis of *Vaha* mRNA level in w^{1118} and *vaha* mutant flies. *Vaha* expression is normalized to *GAPDH*. *Vaha* mRNA is not detected in the mutant. n=3, 10 flies per replicate, data is assessed by two-tailed Student's t-test and presented as mean ± SD. Source data are provided as a source data file.

c Developmental progression of w^{1118}, *vaha* mutants and rescue flies. *vaha* mutants (w^-; *vaha*/*vaha*; +/+) develop slower throughout life and adult mutant flies eclose later than control flies. Gut specific expression of UAS Vaha GFP (w^-; *vaha*/*vaha*.NP1Gal4; UAS Vaha GFP/+) rescues the phenotype.

d 100% of the control flies eclosed by 264 h AEL while mutant flies eclosed by 312 h AEL, a delay of 48 h. Gut specific expression of UAS Vaha GFP rescues the delay. Source data are provided as a source data file.

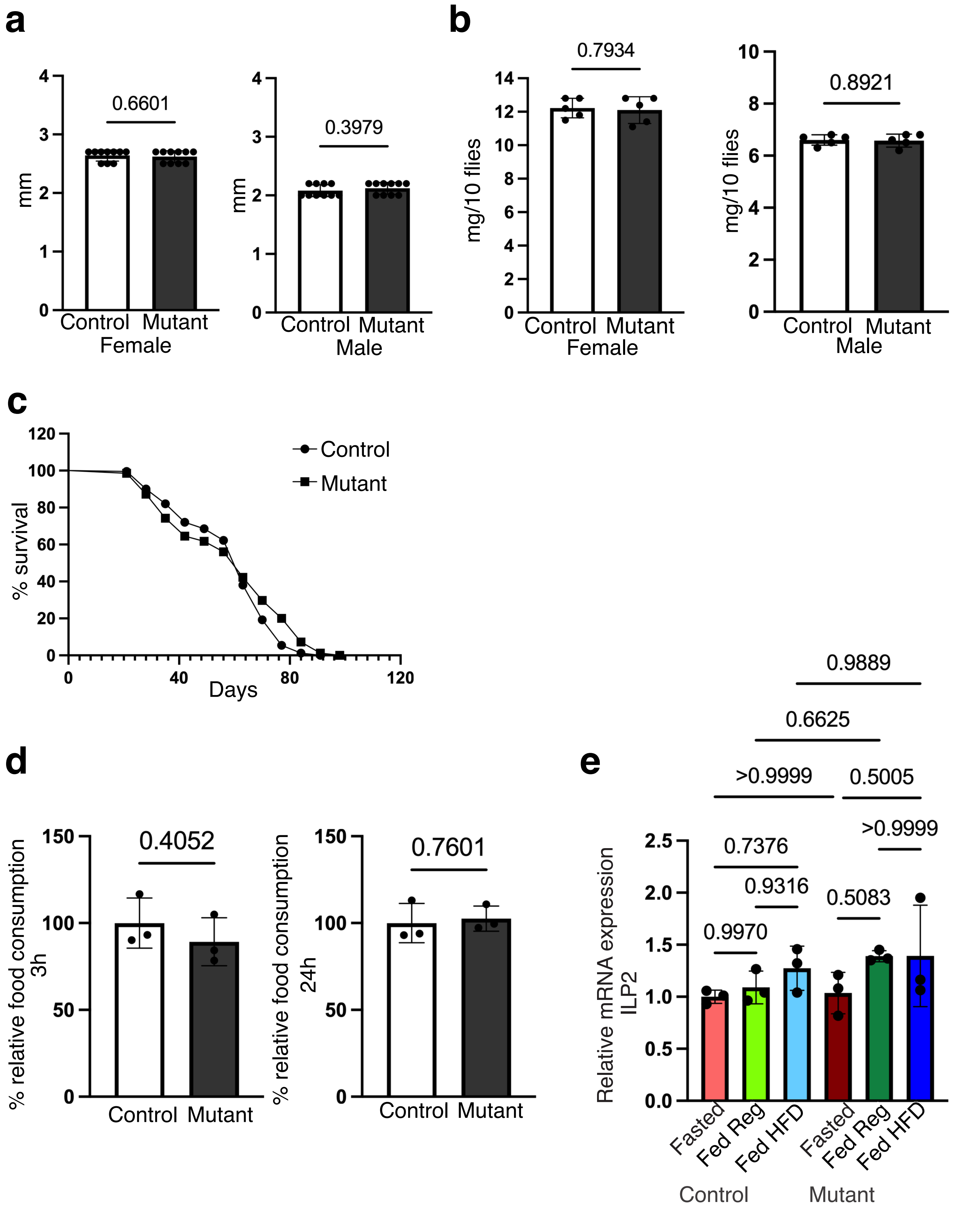

Supplementary Figure 11

Supplementary Fig. 11: *vaha* mutant adult flies are not different in size and weight compared to control and their lifespan is similar to control flies

a The size of *vaha* mutants (female and male) are not different from w^{1118} flies. Each dot represents 1 fly, n=10. Data is assessed by two-tailed Student's t-test and presented as mean ± SD. Source data are provided as a source data file.

b The weight of *vaha* mutants (female and male) are not different from w^{1118} flies. Each dot represents 10 flies, n=5. Data is assessed by two-tailed Student's t-test and presented as mean ± SD. Source data are provided as a source data file.

c Lifespan of control (400 flies) and *vaha* (400 flies) were monitored by transferring them to fresh food every three days for 100 days. Source data are provided as a source data file.

d Capillary feeding (CAFE) assay comparing food intake between control and *vaha* mutant flies. Assays are carried out on 4 day old flies that are starved overnight and then fed for either 3 h or 24 h. n=3, 20 flies per replicate, data is assessed by two-tailed Student's t-test and presented as mean ± SD. Source data are provided as a source data file.

e RT-qPCR analysis of *ILP2* mRNA level in w^{1118} and *vaha* mutant flies fasted overnight and then fed regular or high fat food for 1 h. *ILP2* expression is normalized to *GAPDH*. n=3, 30 heads per replicate, One-way ANOVA followed by Tukey's multiple comparison test is used and data is presented as mean ± SD. Source data are provided as a source data file.

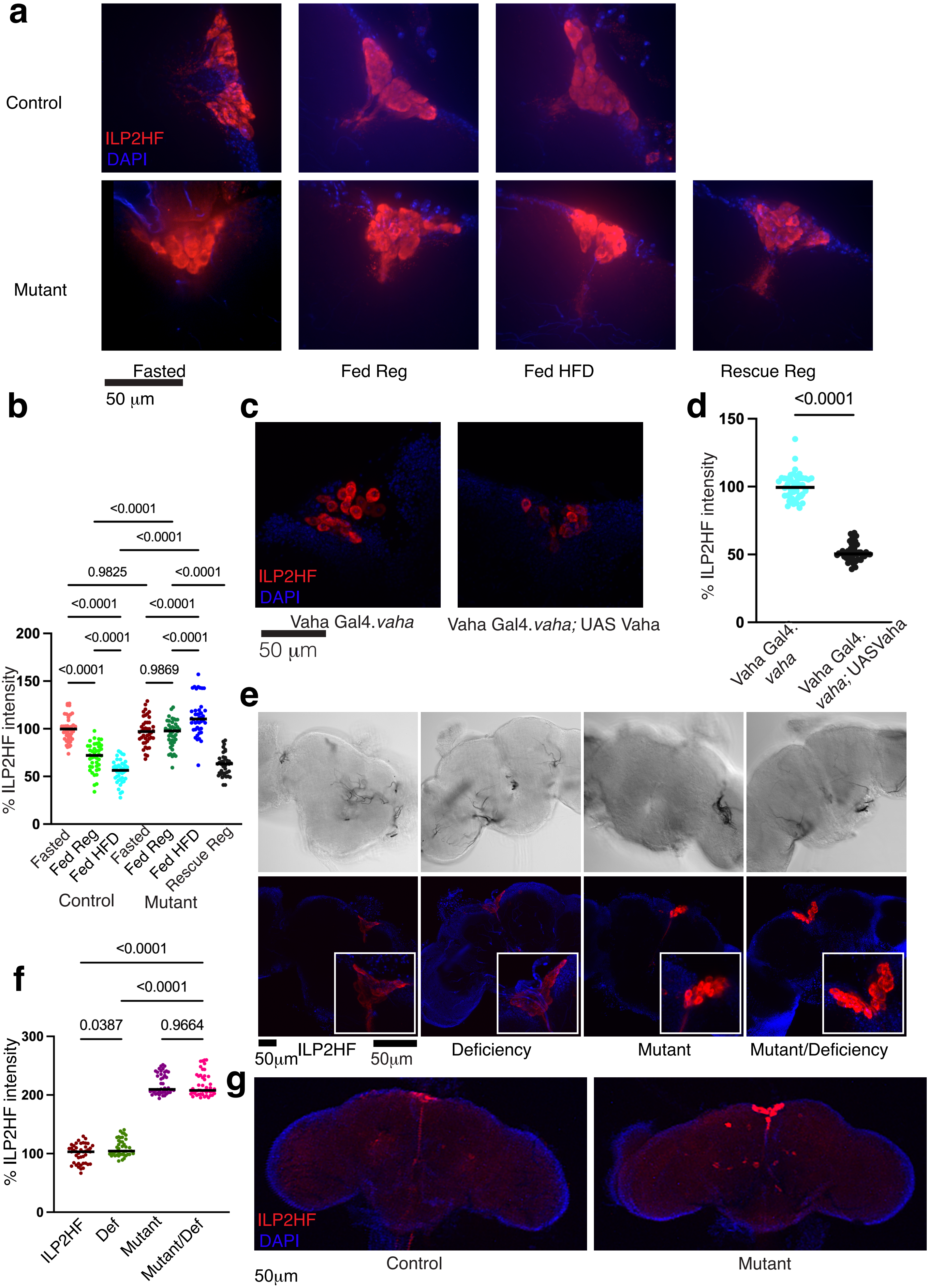

Supplementary Figure 12

Supplementary Fig. 12: *vaha* mutants are defective in ILP2HF secretion

a Immunostaining of brains dissected from 5-7 day old control, *vaha* mutant and rescue flies for ILP2HF (anti HA, red) and DAPI (blue). The genotype of the control flies is (w^-; +/+; gd2HF/gd2HF; *vaha* mutants are w^-; *vaha*/*vaha*; gd2HF/gd2HF and rescue flies are w^-; *vaha*/*vaha*.UASVahaGFP; gd2HF/gd2HF.tubulinGal4. Flies are fasted overnight and fed either regular food (Reg) or regular food containing coconut oil (HFD) for 1 h. ILP2HF staining in the IPCs is more intense in the *vaha* mutant compared to control in the fed state while it is not significantly different in the fasted state. The confocal images are projection of z stacks and images are representative of 6 brains, scale bar 50 μm.

b Quantification of relative ILP2HF fluorescence intensity in the IPCs of control, *vaha* mutants and rescue flies in the fasted and fed states (genotypes and conditions as described in a). Each data point represents fluorescent intensity in one IPC, n=42 (control fasted), n=42 (control fed Reg), n=42 (control fed HFD), n=42 (mutant fasted), n=42 (mutant fed Reg), n=42 (mutant fed HFD), and n=40 (rescue Reg). Data is assessed using One-way ANOVA followed by Tukey's multiple comparison test and the horizontal line indicates median. Source data are provided as a source data file.

c Immunostaining of brains dissected from 5-7 day old *vaha* mutant and rescue flies for ILP2HF (anti HA, red) and DAPI (blue). The genotype of *vaha* mutants are w^-; Vaha Gal4.*vaha*/*vaha*; gd2HF/+ and rescue flies are w^-; Vaha Gal4.*vaha*/*vaha*; gd2HF/UAS Vaha GFP. The confocal images are projection of z stacks and images are representative of 6 brains, scale bar 50 μm.

d Quantification of relative ILP2HF fluorescence intensity in the IPCs of flies described in c. Each data point represents fluorescent intensity in one IPC, n=46 (Vaha Gal4.*vaha* mutant) and n=44 (Vaha Gal4.*vaha;* UASVaha GFP). Data is assessed by two-tailed Student's t-test and horizontal line indicates median. Source data are provided as a source data file.

e Immunostaining of brains dissected from 5-7 day old flies for ILP2HF (anti HA, red) and DAPI (blue). Control flies are w^-; +/+; gd2HF/gd2HF, deficiency flies are w^-; Df(2R)Exel7135/+; gd2HF/gd2HF; *vaha* mutants are w^-; *vaha*/*vaha*; gd2HF/gd2HF and mutant/deficiency are w^-; *vaha*/ Df(2R)Exel7135; gd2HF/gd2HF. The confocal images are projection of z stacks, and the images are representative of 6 brains, scale bar 50 μm.

f Quantification of relative ILP2HF fluorescence intensity in the IPCs of flies described in e. Each data point represents fluorescent intensity in one IPC, n=43 (ILP2HF control), n=43

(deficiency), n=44 (*vaha* mutant) and n=47 (*vaha* mutant/deficiency). One-way ANOVA followed by Tukey's multiple comparison test is used and the horizontal line indicates median. Source data are provided as a source data file.

g Immunostaining of brains dissected from 5-7day old control and *vaha* mutant for ILP2HF (anti HA, red) and DAPI (blue). Control flies are w^-; +/+; gd2HF/gd2HF and *vaha* mutants are w^-; *vaha*/*vaha*; gd2HF/gd2HF. Mutants show increase in ILP2HF staining in the IPCs compared to control. Additionally, some ILP2HF staining is seen in other neurons in the mutants, scale bar 50 µm.

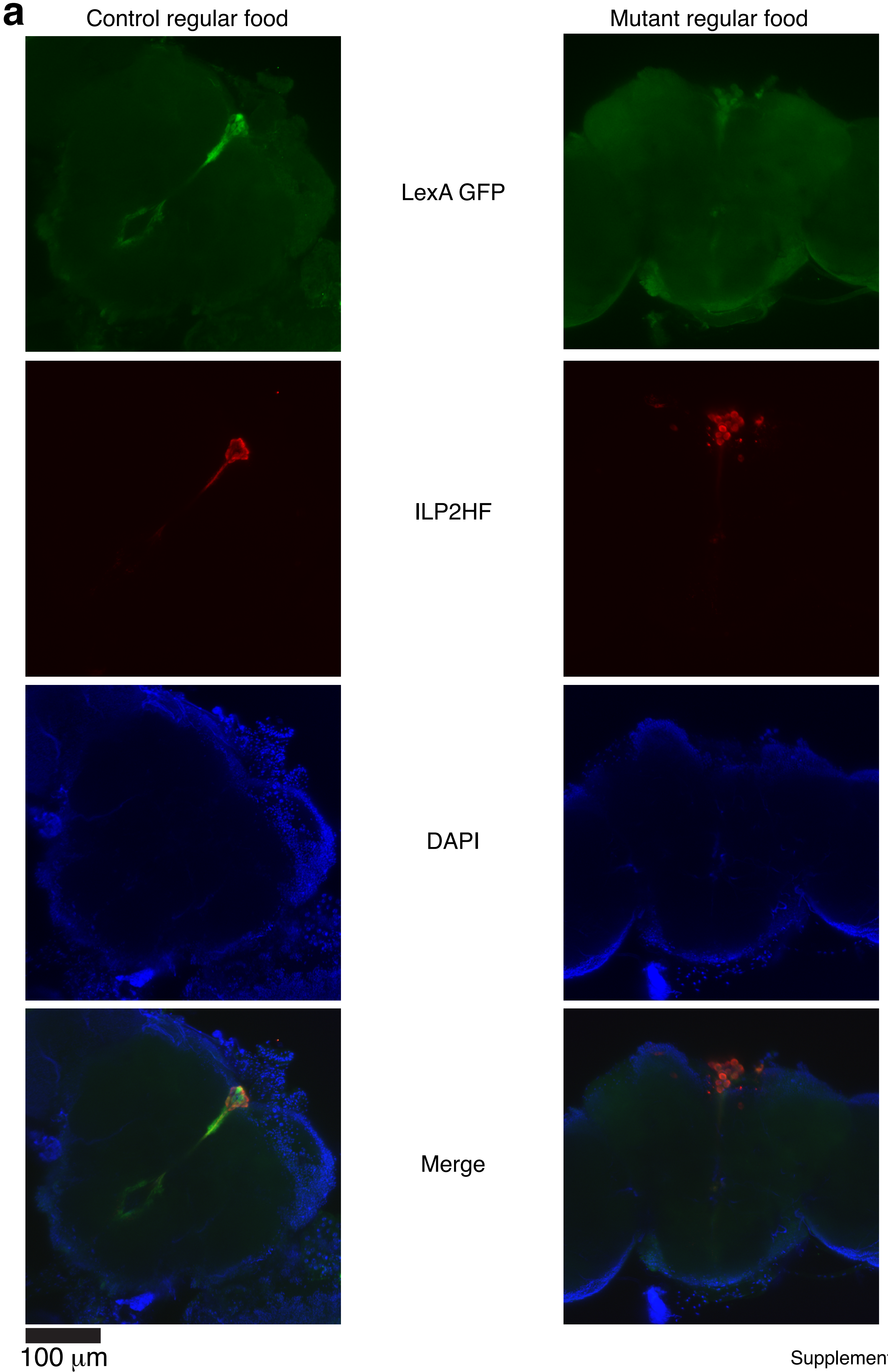

Supplementary Figure 13a

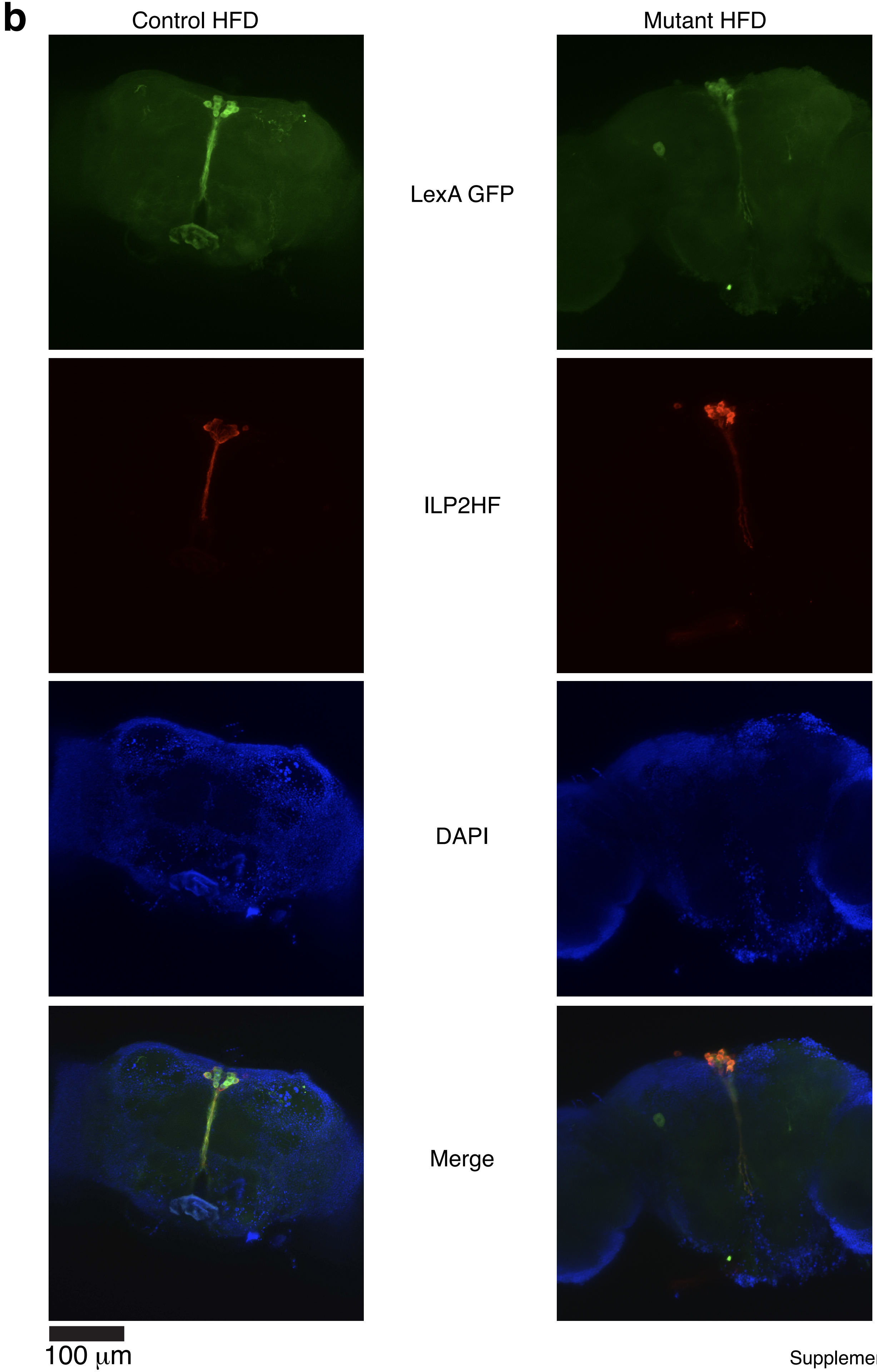

Supplementary Figure 13b

Supplementary Fig. 13: *vaha* mutants show reduced IPC activity compared to control on regular food and HFD

a IPC activity is measured in control and *vaha* mutants on regular food using the CaLexA system. The images show low magnification view of the brain shown in main Fig. 4f (top panel) stained for GFP, ILP2HF, DAPI and the merged images. The confocal images are projection of z stacks and images are representative of 6 brains, scale bar 100 μm.

b IPC activity is measured in control and *vaha* mutants on HFD using the CaLexA system. The images show low magnification view of the brain shown in main Fig. 4f (bottom panel) stained for GFP, ILP2HF, DAPI and the merged images. The confocal images are projection of z stacks and images are representative of 6 brains, scale bar 100 μm.

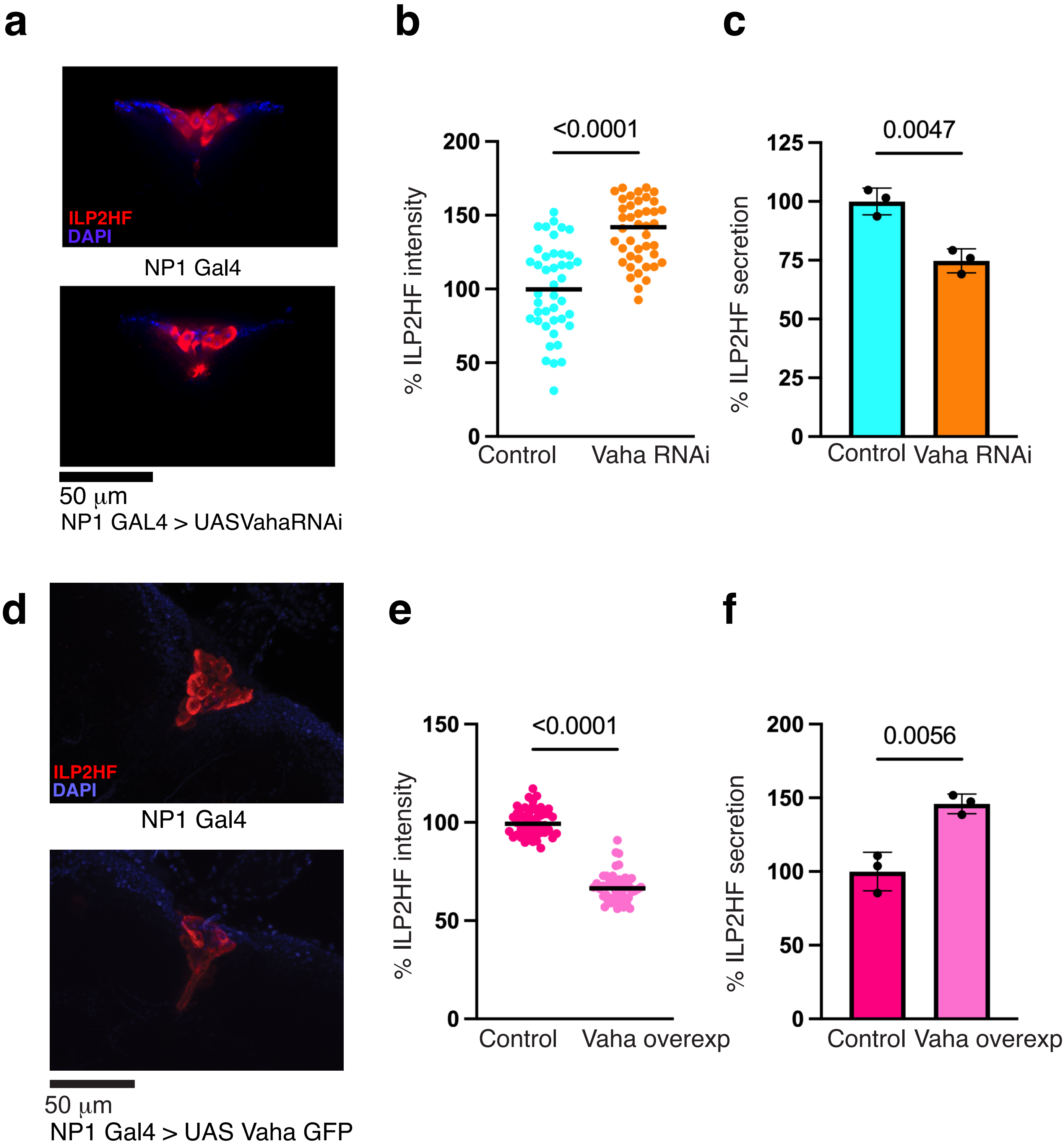

Supplementary Figure 14

Supplementary Fig. 14: Gut specific knockdown of Vaha leads to defect in ILP secretion while overexpression of Vaha leads to increased ILP secretion

a Immunostaining of brains dissected from 5-7day old control or gut specific Vaha RNAi flies for ILP2HF (anti HA, red) and DAPI (blue). The genotype of the control flies is *w*⁻; NP1Gal4/+; +/gd2HF and RNAi flies is *w*⁻; NP1Gal4/+; UASVahaRNAi/gd2HF. ILP2HF staining is significantly more intense in the IPCs of Vaha RNAi flies compared to control. The confocal images are single z sections, and images are representative of 6 brains, scale bar 50 µm.

b Quantification of relative ILP2HF fluorescence intensity in the IPCs of control and gut specific Vaha RNAi flies (genotypes described in a). Each data point represents fluorescent intensity in one IPC, n= 42 (control) and n=42 (Vaha RNAi). Data is assessed by two-tailed Student's t-test and the horizontal line indicates median. Source data are provided as a source data file.

c ILP2HF levels in circulation are assayed in control and Vaha RNAi flies (genotypes as described in d). Circulating ILP2HF levels are lower in the Vaha RNAi compared to control flies. n=3**,** hemolymph from 80 male flies per replicate, data is assessed by two-tailed Student's t-test and presented as mean ± SD. Source data are provided as a source data file.

d Immunostaining of brains dissected from 5-7day old control or gut specific Vaha overexpression flies for ILP2HF (anti HA, red) and DAPI (blue). The genotype of the control flies is *w*⁻; NP1Gal4/+; +/gd2HF and overexpression flies is *w*⁻; NP1Gal4/+; UASVahaGFP/gd2HF. ILP2HF staining in the IPCs is reduced in the overexpressors compared to control. The data from control flies in Figs. 4a and 4b are also used here as control since these experiments were performed at the same time. The confocal images are projection of z stacks, and images are representative of 6 brains, scale bar 50 µm.

e Quantification of relative ILP2HF fluorescence intensity in the IPCs of control and gut specific Vaha overexpression flies (genotypes described in d). Each data point represents fluorescent intensity in one IPC, n= 51 (control) and n=50 (Vaha overexp). Data is assessed by two-tailed Student's t-test and the horizontal line indicates median. Source data are provided as a source data file.

f ILP2HF levels in circulation were assayed in control and Vaha overexpression flies (genotypes as described in d). Circulating ILP2HF levels are higher in the Vaha overexpressors compared to control flies. n=3**,** 15 male flies per replicate, data is assessed by two-tailed Student's t-test and presented as mean ± SD. Source data are provided as a source data file.

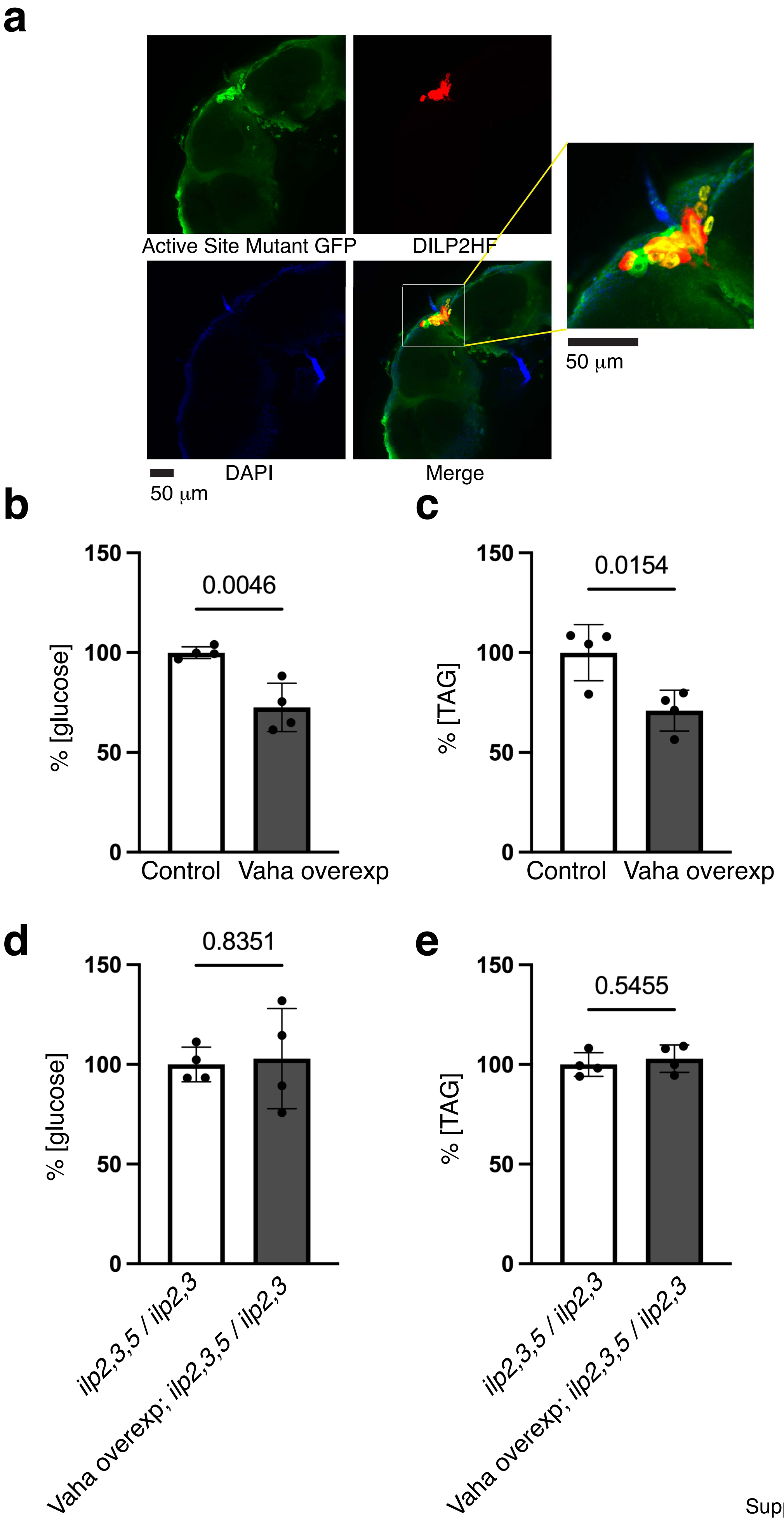

Supplementary Figure 15

Supplementary Fig. 15: Gut specific overexpression of Vaha reduces whole body glucose and TAG

a Immunostaining for active site mutant GFP (anti GFP, green) and ILP2HF (anti HA, red) in brains dissected from 5-7day old adult flies expressing Vaha active site mutant GFP in the gut and ILP2HF under the control of its own promoter. In the active site transgenic, the Ser-His-Asp catalytic triad required for lipase activity are replaced by Ala. Nuclei are stained with DAPI (blue). A higher magnification image of the boxed region in the merged panel is shown. There is considerable overlap of GFP and HA staining in the IPCs indicating that lipase activity is not required for Vaha movement from the gut to the brain. The confocal images are projection of z stacks, and the images are representative of 6 brains, scale bar 50 μm.

b Glucose content is measured in homogenates from control *w*$^-$; NP1Gal4/+; gd2HF/+ and overexpression flies *w*$^-$; NP1Gal4/+; UASVahaGFP/gd2HF flies (fasting followed by feeding). Each data point represents value from 10 flies, n=4, data is assessed by two-tailed Student's t-test and presented as mean ± SD. Source data are provided as a source data file.

c TAG content is measured in homogenates from flies (fasting followed by feeding) described in b. Each data point represents value from 10 flies, n=4, data is assessed by two-tailed Student's t-test and presented as mean ± SD. Source data are provided as a source data file.

d Glucose content is measured in insulin mutant flies and insulin mutant flies in which Vaha is overexpressed in the gut. The genotypes of flies are *w*$^-$; NP1Gal4/+; *ilp2,3,5/ilp2,3* and *w*$^-$; NP1Gal4/UAS VahaGFP; *ilp2,3,5/ilp2,3*. Each dot represents value from 10 flies, n=4, data is assessed by two-tailed Student's t-test and presented as mean ± SD. Source data are provided as a source data file.

e TAG content is measured in flies described in d. Each dot represents value from 10 flies, n=4, data is assessed by two-tailed Student's t-test and presented as mean ± SD. Source data are provided as a source data file.

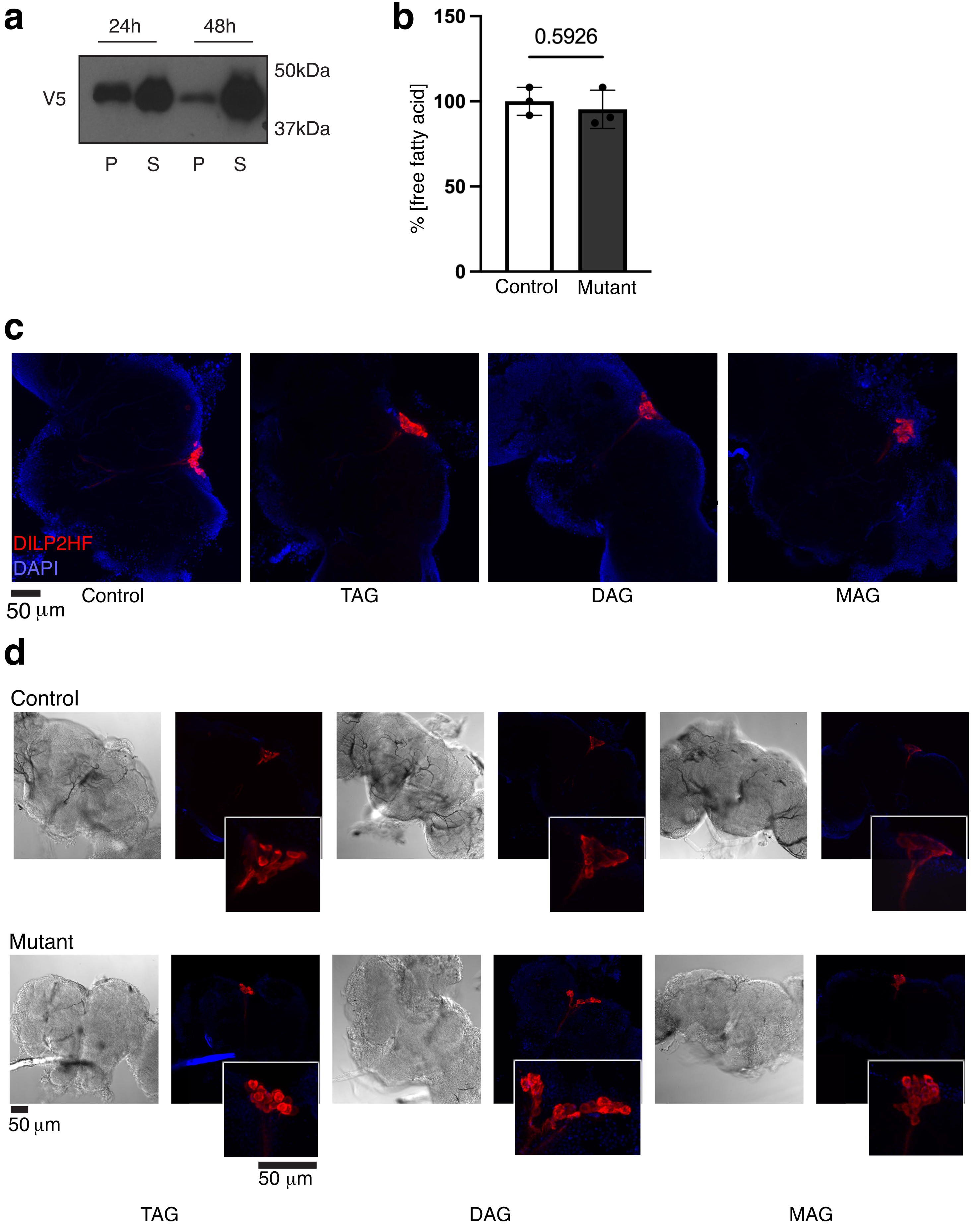

Supplementary Figure 16

Supplementary Fig. 16: Dietary supplementation of lipids increases ILP2HF release from IPCs

a Immunoblotting of cell pellet (P) and culture supernatant (S) from stable S2 cells expressing Vaha V5. The cells are induced with copper sulfate for 24 h or 48 h, extracts prepared from the cell pellet and culture medium and Western blotting is carried out with V5 antibody. Robust Vaha V5 expression in the supernatant fraction shows it is a secreted protein in S2 cells as well. n=3. Source data are provided as a source data file.

b Free fatty acid levels are estimated in whole fly homogenates of 30-35day old control and *vaha* mutant flies by UPLC-MS/MS. Free fatty acid levels are not significantly different in the *vaha* mutant compared to control flies. Data is obtained from three biological replicates with100 flies per replicate. Data is assessed by two-tailed Student's t-test and presented as mean ± SD. Source data are provided as a source data file.

c Immunostaining of brains dissected from 5-7day old control flies (w^-; +/+; gd2HF/gd2HF) and flies fed either TAG, DAG, or MAG. Flies are starved overnight and then transferred to regular food or regular food supplemented with either TAG, DAG, or MAG for 2-3h. Brains are dissected and immunostained for ILP2HF (anti HA, red) and DAPI (blue). There is less ILP2HF staining in the IPCs in lipid supplemented food compared to control. The confocal images are projection of z stacks, and the images are representative of 6 brains, scale bar 50 µm.

d Immunostaining of brains dissected from 5-7day old control flies (w^-; +/+; gd2HF/gd2HF) or vaha mutant flies (w^-; *vaha*/*vaha*; gd2HF/gd2HF) fed either TAG, DAG, or MAG. Flies are starved overnight and then transferred to regular food or regular food supplemented with either TAG, DAG, or MAG for 2-3h. Brains are dissected and immunostained for ILP2HF (anti HA, red) and DAPI (blue). The confocal images are projection of z stacks, and the images are representative of 6 brains, scale bar 50 µm.

Supplementary Table 1

Metabolites	Pathways	Pathology
↓ Gamma–glutamylthreonine	Glutathione metabolism	Diabetes specific coronary heart disease[1,2]
↓ Citrulline	Arginine metabolism	Endothelial dysfunction → vascular complications in diabetes[3]
↓ 3–hydroxykynurenine	Tryptophan metabolism	Cognitive decline[4]
↓ Carotene diol	Carotenoid metabolism	Decrease associated with increased fasting glycemia[5]
↓ Alpha–ketoglutarate	TCA cycle	Decrease associated with decreased GSIS[6]
↓ 2–oxoarginine	Arginine metabolism	Endothelial dysfunction → vascular complications in diabetes[7]
↓ 3–methyl–2–oxovalerate coupled with ↑ 3–hydroxybutyrylcarnitine	Branched chain amino acid catabolism	Impaired fasting glucose[8]
↑ 6–phosphogluconate	Pentose phosphate pathway	Increase and decrease associated with defective GSIS[9]
↑ Aspartate	Non-essential amino acid metabolism	Plasma concentration increased in T2D[10]

Supplementary Table 1: Metabolites strongly associated with diabetes progression that show changes in *vaha* mutant

The metabolites from the heat map in Fig. 7d are manually reviewed for known metabolic signatures and biomarkers of diabetes in human subjects. The decrease or increase in metabolite, the affected pathway and associated pathophysiology is listed.

Lipid species	Control 1	Control 2	Control 3	Mutant 1	Mutant 2	Mutant 3	Fold change Mutant/Control	P-value
DAG(12:0/16:0)	1.1510	0.9680	0.9129	0.9554	1.0067	1.5844	1.17	0.3046
DAG(12:0/18:0)	1.0520	1.0028	0.8799	0.9930	1.0032	1.0696	1.04	0.6501
DAG(12:0/18:1)	1.0139	1.0010	0.9503	1.0688	1.1631	1.4926	1.26	0.0537
DAG(12:0/18:2)	1.1736	1.4637	1.2539	0.8191	0.9113	1.0144	0.71	0.0858
DAG(14:0/14:0)	1.5829	1.3681	1.2967	1.2143	1.2506	1.5991	0.96	0.5363
DAG(14:0/16:0)	1.0889	0.9573	1.0174	1.2160	1.2041	1.7293	1.35	0.0092
DAG(14:0/16:1)	2.0662	1.9145	2.1293	2.2377	2.0808	3.0227	1.2	0.353
DAG(14:0/18:1)	0.8960	0.8699	0.7912	1.5340	1.5052	1.9542	1.95	0.0001
DAG(14:0/18:2)	1.2080	1.4918	1.3600	1.2212	1.0981	1.4320	0.92	0.6635
DAG(14:0/18:3)	1.4065	1.6641	1.4893	1.7626	1.2970	1.6698	1.04	0.88
DAG(14:0/20:0)	1.1835	0.9070	0.8603	1.3858	1.3092	1.6845	1.48	0.0029
DAG(14:0/20:4)	0.6720	1.2811	1.0000	0.6720	0.6720	0.6720	0.68	0.1666
DAG(14:0/22:6)	0.7979	1.1490	0.8356	0.8904	0.7671	1.2611	1.05	0.8547
DAG(14:1/16:0)	1.3163	1.0257	0.9590	1.7278	1.6083	2.1657	1.67	<0.0001
DAG(14:1/18:1)	0.8365	0.9100	0.6692	1.2283	1.1497	1.2748	1.51	0.0031
DAG(15:0/18:1)	0.9980	0.8823	1.0046	0.7517	0.5619	0.6699	0.69	0.0224
DAG(15:0/18:2)	1.0502	1.0061	1.1301	0.7139	0.7876	0.6460	0.67	0.0023
DAG(16:0/16:0)	0.7112	0.6773	0.6127	1.1339	1.0863	1.5665	1.89	<0.0001
DAG(16:0/16:1)	0.8430	0.7950	0.7634	1.3808	1.2385	1.9786	1.91	<0.0001
DAG(16:0/18:0)	0.5264	0.6616	0.5401	0.8582	0.8256	1.0617	1.59	0.0096
DAG(16:0/18:1)	0.5173	0.5184	0.4092	1.1603	1.0615	1.4526	2.54	0.0002
DAG(16:0/18:2)	0.7503	0.8812	0.7076	0.9228	0.8095	1.0080	1.17	0.068
DAG(16:0/18:3)	0.8561	0.8856	0.8097	0.8928	0.9948	1.1310	1.18	0.06
DAG(16:0/20:3)	1.1134	0.6585	1.0274	0.6585	1.3591	0.6585	0.96	0.682
DAG(16:0/20:4)	0.7411	1.4714	0.7411	0.7411	1.6065	0.7411	1.05	0.8922
DAG(16:0/20:5)	0.7347	0.7347	0.7347	0.7347	0.7347	0.7347	1	1
DAG(16:0/22:5)	2.2324	0.8884	1.1775	0.6167	1.0196	0.9617	0.6	0.1247
DAG(16:0/22:6)	0.8333	0.7145	0.7145	0.7145	0.8217	0.7145	0.99	0.977
DAG(16:1/16:1)	1.0025	1.0407	0.9412	1.5006	1.4888	1.6758	1.56	<0.0001
DAG(16:1/18:0)	1.1241	1.0008	1.1072	1.1321	1.0275	1.5256	1.14	0.2264
DAG(16:1/18:1)	0.7026	0.7263	0.6499	1.1686	1.1149	1.2945	1.72	0.0002
DAG(16:1/18:2)	1.0296	1.3272	1.0655	0.9699	0.8885	1.0196	0.84	0.0592
DAG(16:1/18:3)	1.0581	1.2176	0.9669	0.8908	1.0998	1.0854	0.95	0.5353
DAG(16:1/20:0)	1.2283	1.1903	1.1073	1.8745	1.6988	1.9152	1.56	0.0022
DAG(16:1/20:2)	1.3222	1.1117	1.5409	1.1480	1.0807	1.1436	0.85	0.1777
DAG(16:1/20:4)	1.3414	0.7250	0.7250	0.7250	0.7250	0.7250	0.78	0.3443
DAG(16:1/22:6)	1.3161	1.5322	1.3103	0.6908	1.0264	0.6908	0.58	0.0058
DAG(18:0/18:1)	0.8124	0.7904	0.7792	0.8335	0.7398	1.1547	1.15	0.4559
DAG(18:0/18:2)	1.1751	1.1969	1.0710	0.8914	0.6968	0.8055	0.7	0.0015
DAG(18:0/18:3)	1.3871	1.4260	1.1744	1.1652	0.9358	0.9240	0.76	0.0042
DAG(18:0/22:6)	1.1206	1.2169	0.8528	0.9234	0.9335	0.9785	0.89	0.5495

Lipid species	Control 1	Control 2	Control 3	Mutant 1	Mutant 2	Mutant 3	Fold change Mutant/Control	P-value
DAG(18:1/18:1)	0.5155	0.5719	0.4975	0.7590	0.7038	0.8975	1.49	0.0265
DAG(18:1/18:2)	0.8933	1.1486	0.8870	0.7668	0.7037	0.8393	0.79	0.0165
DAG(18:1/20:1)	0.7335	0.9114	0.7463	0.9170	1.1137	1.0840	1.3	0.035
DAG(18:1/20:2)	1.3799	0.9531	1.0121	1.0089	1.0215	0.8970	0.88	0.4643
DAG(18:1/20:3)	1.5346	0.9558	1.3177	0.8715	0.9989	0.9781	0.75	0.2342
DAG(18:1/20:4)	0.8738	0.8258	0.9625	0.8258	0.8258	0.8258	0.93	0.3211
DAG(18:1/20:5)	0.6808	0.6808	1.0635	0.6808	0.6808	0.6808	0.84	0.1089
DAG(18:1/22:4)	1.5383	1.0095	1.0688	0.9345	1.1973	0.9527	0.85	0.4125
DAG(18:1/22:5)	1.4837	0.7928	0.7928	0.7928	0.7928	0.7928	0.77	0.1499
DAG(18:1/22:6)	0.5172	0.9401	0.8624	1.1265	1.0166	0.8690	1.3	0.3013
DAG(18:2/18:3)	0.9943	1.3047	1.1363	0.8825	1.1261	0.9578	0.86	0.3122
DAG(18:2/20:3)	1.2219	1.0004	1.0664	1.0441	1.1051	0.9490	0.94	0.5555
DAG(18:2/20:4)	0.7804	0.7804	0.7804	0.7804	0.7804	0.7804	1	1
DAG(18:2/20:5)	0.7250	0.7250	0.7250	0.7250	0.8578	0.7250	1.06	0.5177
DAG(18:2/22:4)	1.5648	1.1657	1.1536	1.0459	1.0535	0.5151	0.67	0.032
DAG(18:2/22:5)	0.6400	0.6400	0.9465	0.6400	0.6400	1.7161	1.35	0.4597
DAG(18:2/22:6)	1.1790	1.0727	0.6945	0.6249	0.6249	0.6249	0.64	0.0216
Total DAG	1.0675	1.0185	0.9925	1.3189	1.2574	1.7012	1.39	0.0003
MAG(12:0)	1.5446	1.3477	1.3235	1.0851	0.9531	1.0469	0.73	0.0535
MAG(14:0)	1.5735	1.4640	1.4537	0.8745	0.9680	0.9344	0.62	0.0002
MAG(14:1)	2.9566	2.3212	1.9498	1.3910	1.3821	1.0326	0.53	0.0042
MAG(15:0)	0.8714	1.2129	1.0625	1.0856	0.9193	1.1641	1.01	0.9073
MAG(16:0)	0.8962	1.0532	1.0937	0.5445	0.6147	0.6240	0.59	0.0372
MAG(16:1)	1.8362	1.5890	1.8811	1.1681	1.0345	0.9780	0.60	0.0001
MAG(17:0)	1.1598	1.4052	1.3502	0.7962	0.6666	0.6544	0.54	0.0098
MAG(18:0)	0.6959	1.0320	1.0987	0.5277	0.5968	0.5737	0.60	0.1050
MAG(18:1)	1.1237	1.2945	1.2295	1.0451	0.8570	0.9438	0.78	0.0166
MAG(18:2)	1.6483	1.6099	1.3605	1.0525	0.8226	0.9459	0.61	0.0180
MAG(18:3)	1.3613	1.1010	0.9565	1.1607	1.0508	1.0743	0.96	0.8411
MAG(18:4)	1.1237	0.8282	0.7117	0.7117	0.7117	0.7117	0.80	0.1467
MAG(20:0)	1.0545	1.2216	0.9323	0.8603	0.7681	0.9997	0.82	0.4370
MAG(20:1)	0.8997	1.0000	0.8829	1.1123	1.4797	1.0995	1.33	0.2587
MAG(20:2)	0.7208	0.7208	0.9891	0.7208	0.7208	0.8561	0.95	0.8160
MAG(20:3)	0.9566	0.9566	0.9566	0.9566	0.9566	0.9566	1.00	1.0000
MAG(20:4)	1.0000	1.0000	1.0000	1.0000	1.0000	1.0000	1.00	1.0000
MAG(20:5)	1.3539	1.5043	0.9138	0.9013	0.6105	0.8589	0.63	0.0572
MAG(22:0)	0.9187	0.9353	0.9433	0.8512	0.5419	1.2027	0.93	0.4782
MAG(22:1)	0.8074	1.0451	0.7614	0.7614	0.9609	0.7658	0.95	0.7371
MAG(22:2)	0.7510	0.7510	0.7510	0.7510	0.7510	0.7510	1.00	1.0000
MAG(22:5)	0.6823	0.6823	0.6823	0.6823	0.6823	0.6823	1.00	1.0000
MAG(22:6)	0.6699	1.0864	0.6699	0.6699	0.6699	0.6699	0.83	0.5120

Lipid species	Control 1	Control 2	Control 3	Mutant 1	Mutant 2	Mutant 3	Fold change Mutant/Control	P-value
MAG(24:0)	1.1181	1.0971	0.9443	0.5805	0.9130	0.9845	0.78	0.2381
MAG(24:1)	0.6752	0.6752	0.6752	0.6752	0.8988	0.6752	1.11	0.6080
Total MAG	0.9796	1.1412	1.1554	0.7048	0.7031	0.7202	0.65	0.024

Supplementary Table 2

Supplementary Table 2: List of DAG and MAG species profiled in control and *vaha* mutant

The table lists the molecular species of DAG and MAG that are profiled in control and *vaha* mutant, the p value and fold change (mutant/control). Welch's two sample *t*-test is used to assess biochemicals that differed significantly between the two groups. 3 biological replicates are performed with 100 flies per replicate.

Supplementary References

1.Zaghlool, S.B., Halama, A., Stephan, N., Thangam, M., Ahlqvist, E., Albagha, O.M.E., Samra, A.B.A., & Suhre. Metabolic and proteomic signatures of type 2 diabetes subtypes in an Arab population. medRxiv doi: https://doi.org/10.1101/2022.01.13.22269204.

2. Pipino, C., Shah, H., Prudente, S., Di Pietro, N., Zeng, L., Park, K., Trischitta, V., Pennathur, S., Pandolfi, A., & Doria, A. Association of the 1q25 Diabetes-Specific Coronary Heart Disease Locus with Alterations of the γ-Glutamyl Cycle and Increased Methylglyoxal Levels in Endothelial Cells. *Diabetes* **69**, 2206-2216 (2020).

3. Kövamees, O., Shemyakin, A., & Pernow, J. Amino acid metabolism reflecting arginase activity is increased in patients with type 2 diabetes and associated with endothelial dysfunction. *Diab Vasc Dis Res*. **13**, 354-360 (2016).

4. Bakker, L., Ramakers, I.H.G.B., van Boxtel, M.P.J., Schram, M.T., Stehouwer, C.D.A., van der Kallen, C.J.H., Dagnelie, P.C., van Greevenbroek, M.M.J., Wesselius, A., Midttun, Ø., Ueland, P.M., Verhey, F.R.J., Eussen, S.J.P.M., & Köhler, S. Associations between plasma kynurenines and cognitive function in individuals with normal glucose metabolism, prediabetes and type 2 diabetes: the Maastricht Study. *Diabetologia* **64**, 2445-2457 (2021).

5. Coyne, T., Ibiebele, T.I., Baade, P.D., Dobson, A., McClintock, C., Dunn, S., Leonard, D., & Shaw, J. Diabetes mellitus and serum carotenoids: findings of a population-based study in Queensland, Australia. *Am J Clin Nutr.* 82, 685-693 (2005).

6. Yuan, Y., Zhu, C., Wang, Y., Sun, J., Feng, J., Ma, Z., Li, P., Peng, W., Yin, C., Xu, G., Xu, P., Jiang, Y., Jiang, Q., & Shu, G. α-Ketoglutaric acid ameliorates hyperglycemia in diabetes by inhibiting hepatic gluconeogenesis via serpina1e signaling. *Sci Adv.* **8**, 18 eabn2879 (2022).

7. Piatti, P.M., Monti, L.D., Valsecchi, G., Magni, F., Setola, E., Marchesi, F., Galli-Kienle, M., Pozza, G., & Alberti, K.G. Long-term oral L-arginine administration improves peripheral and hepatic insulin sensitivity in type 2 diabetic patients. *Diabetes Care* **24**, 875-880 (2001).

8. Menni, C., Fauman, E., Erte, I., Perry, J.R., Kastenmüller, G., Shin, S.Y., Petersen, A.K., Hyde, C., Psatha, M., Ward, K.J., Yuan, W., Milburn, M., Palmer, C.N., Frayling, T.M., Trimmer, J., Bell, J.T., Gieger, C., Mohney, R.P., Brosnan, M.J., Suhre, K., Soranzo, N., & Spector, T.D. Biomarkers for type 2 diabetes and impaired fasting glucose using a nontargeted metabolomics approach. *Diabetes* **62**, 4270-4276 (2013).

9. Palomino-Schätzlein, M., Lamas-Domingo, R., Ciudin, A., Gutiérrez-Carcedo, P., Marés, R., Aparicio-Gómez, C., Hernández, C., Simó, R., & Herance, J.R. A Translational In Vivo and In Vitro Metabolomic Study Reveals Altered Metabolic Pathways in Red Blood Cells of Type 2 Diabetes. *J Clin Med.* **9**, 1619 (2020).

10. Alqudah, A., Wedyan, M., Qnais, E., Jawarneh, H., & McClements, L. Plasma Amino Acids Metabolomics' Important in Glucose Management in Type 2 Diabetes. Front Pharmacol. 12, doi: 10.3389/fphar.2021.695418 (2021).
