# Supplementary material for: A nutrient responsive lipase mediates gut-brain communication to regulate insulin secretion in Drosophila
Source: Nat Commun. 2024 May 23;15:4410. doi: 10.1038/s41467-024-48851-8 (PMC11116528; doi:10.1038/s41467-024-48851-8)
Supplement: Supplementary file 3 — Description of Additional Supplementary Files [file 41467_2024_48851_MOESM3_ESM.pdf]

Description of Additional Supplementary Dataset for:

A nutrient responsive lipase mediates gut-brain communication to regulate insulin secretion in *Drosophila*

Alka Singh[1+], Kandahalli Venkataranganayaka Abhilasha [2+], Kathya R. Acharya[1,2,4], Haibo Liu[1], Niraj K. Nirala[3], Velayoudame Parthibane[2], Govind Kunduri[2], Thiruvaimozhi Abimannan[2], Jacob Tantalla[2], Lihua Julie Zhu[1], Jairaj K. Acharya[2*] and Usha R. Acharya[2*]

Additional Supplementary Dataset include Supplementary Data1, Supplementary Data 2, Supplementary Data 3 and Supplementary Data 4 as Excel files and accompanying legends.

Supplementary Data 1: List of lipids that were profiled, their pathways and fold change (mutant/control) are shown.

Lipids are analyzed by UPLC-MS/MS. 3 biological replicates were performed with 100 flies per replicate. Welch's two sample *t*-test is used to assess lipids that differed significantly between the two groups. p value of 0.05 or less and q value (false discovery rate) of 0.1 or less are considered statistically significant. Red denotes increase while green denotes decrease in the mutant compared to control. The species approaching significance ($0.05<p<0.10$) are shown in pink (increase) and light green (decrease).

Supplementary Data 2: List of biochemicals that were profiled, the pathways they belong to, and fold change (mutant/control) are shown.

The metabolites are analyzed by UPLC-MS/MS. 3 biological replicates are performed with 100 flies per replicate. Welch's two sample *t*-test is used to assess biochemicals that differed significantly between the two groups. p value of 0.05 or less and q value (false discovery rate) of 0.1 or less are considered statistically significant. Red denotes increase while green denotes decrease in the mutant compared to control. The biochemicals approaching significance ($0.05<p<0.10$) are shown in pink (increase) and light green (decrease).

Supplementary Data 3: Raw data that was used to generate the fold change (mutant/control) of lipids shown in Supplementary Data 1.

30-35day old flies were used for analyses. 3 biological replicates were performed with 100 flies per replicate.

Supplementary Data 4: Raw data that was used to generate the fold change (mutant/control) of metabolites shown in Supplementary Data 2.

30-35day old flies were used for analyses. 3 biological replicates were performed with 100 flies per replicate.
