# Supplementary material for: A nutrient responsive lipase mediates gut-brain communication to regulate insulin secretion in Drosophila
Source: Nat Commun. 2024 May 23;15:4410. doi: 10.1038/s41467-024-48851-8 (PMC11116528; doi:10.1038/s41467-024-48851-8)
Supplement: Supplementary file 8 — Reporting Summary [file 41467_2024_48851_MOESM8_ESM.pdf]

nature portfolio

Corresponding author(s): Usha R. Acharya; Jairaj K. Acharya

Last updated by author(s): May 1, 2024

Reporting Summary

Nature Portfolio wishes to improve the reproducibility of the work that we publish. This form provides structure for consistency and transparency in reporting. For further information on Nature Portfolio policies, see our Editorial Policies and the Editorial Policy Checklist.

Statistics

For all statistical analyses, confirm that the following items are present in the figure legend, table legend, main text, or Methods section.

n/a	Confirmed	
☐	☒	The exact sample size (*n*) for each experimental group/condition, given as a discrete number and unit of measurement
☐	☒	A statement on whether measurements were taken from distinct samples or whether the same sample was measured repeatedly
☐	☒	The statistical test(s) used AND whether they are one- or two-sided<br>*Only common tests should be described solely by name; describe more complex techniques in the Methods section.*
☒	☐	A description of all covariates tested
☐	☒	A description of any assumptions or corrections, such as tests of normality and adjustment for multiple comparisons
☐	☒	A full description of the statistical parameters including central tendency (e.g. means) or other basic estimates (e.g. regression coefficient) AND variation (e.g. standard deviation) or associated estimates of uncertainty (e.g. confidence intervals)
☐	☒	For null hypothesis testing, the test statistic (e.g. *F*, *t*, *r*) with confidence intervals, effect sizes, degrees of freedom and *P* value noted<br>*Give P values as exact values whenever suitable.*
☒	☐	For Bayesian analysis, information on the choice of priors and Markov chain Monte Carlo settings
☒	☐	For hierarchical and complex designs, identification of the appropriate level for tests and full reporting of outcomes
☒	☐	Estimates of effect sizes (e.g. Cohen's *d*, Pearson's *r*), indicating how they were calculated

Our web collection on statistics for biologists contains articles on many of the points above.

Software and code

Policy information about availability of computer code

Data collection	No unpublished software was used in data collection. Confocal images were acquired on a Zeiss LSM 880 microscope, Nikon spinning disk confocal microscope, and Leica-Andor spinning disk confocal microscope. Metabolomics and lipidomics were performed using ultrahigh performance liquid chromatography-tandem mass spectrometry by Metabolon.
Data analysis	No unpublished code was used in data analysis. Metabolomics data analyses were carried out using R (version 4.0). Smooth quantile normalization was performed as in PMID:29036413. Differential abundance analysis was performed using Limma package (PMID:25605792). The nominal p values were adjusted using the Benjamini-Hochberg method (https://www.jstor.org/stable/2346101). Heatmaps were generated using the pheatmap package. All other statistical analysis was by GraphPad Prism 10 software. Confocal image analysis was performed using Fiji.

For manuscripts utilizing custom algorithms or software that are central to the research but not yet described in published literature, software must be made available to editors and reviewers. We strongly encourage code deposition in a community repository (e.g. GitHub). See the Nature Portfolio guidelines for submitting code & software for further information.

Data

Policy information about availability of data

All manuscripts must include a data availability statement. This statement should provide the following information, where applicable:

- Accession codes, unique identifiers, or web links for publicly available datasets
- A description of any restrictions on data availability
- For clinical datasets or third party data, please ensure that the statement adheres to our policy

The raw data for lipidomics and metabolomics has been uploaded to Metabolights with accession number MTBLS8441. The analyzed data is provided in Supplementary Tables 1 and 2 in the manuscript.

Human research participants

Policy information about studies involving human research participants and Sex and Gender in Research.

Reporting on sex and gender	*Use the terms sex (biological attribute) and gender (shaped by social and cultural circumstances) carefully in order to avoid confusing both terms. Indicate if findings apply to only one sex or gender; describe whether sex and gender were considered in study design whether sex and/or gender was determined based on self-reporting or assigned and methods used. Provide in the source data disaggregated sex and gender data where this information has been collected, and consent has been obtained for sharing of individual-level data; provide overall numbers in this Reporting Summary. Please state if this information has not been collected. Report sex- and gender-based analyses where performed, justify reasons for lack of sex- and gender-based analysis.*
Population characteristics	*Describe the covariate-relevant population characteristics of the human research participants (e.g. age, genotypic information, past and current diagnosis and treatment categories). If you filled out the behavioural & social sciences study design questions and have nothing to add here, write "See above."*
Recruitment	*Describe how participants were recruited. Outline any potential self-selection bias or other biases that may be present and how these are likely to impact results.*
Ethics oversight	*Identify the organization(s) that approved the study protocol.*

Note that full information on the approval of the study protocol must also be provided in the manuscript.

Field-specific reporting

Please select the one below that is the best fit for your research. If you are not sure, read the appropriate sections before making your selection.

☒ Life sciences ☐ Behavioural & social sciences ☐ Ecological, evolutionary & environmental sciences

For a reference copy of the document with all sections, see nature.com/documents/nr-reporting-summary-flat.pdf

Life sciences study design

All studies must disclose on these points even when the disclosure is negative.

Sample size	Sample size was chosen based on published literature and historical precedence.
Data exclusions	Data were not excluded from analyses
Replication	Results were verified using independent biological replicates. Mutant flies were isogenized by backcrossing. Age matched control and mutant flies were used for each replicate. For imaging experiments, at least 6 brains and 6 guts were analyzed for each condition. For Western blotting, each experiment was replicated thrice. For qPCR experiments, each experiment was performed in duplicate and replicated three times. For enzyme assays, each substrate concentration was analyzed three times in duplicate. For metabolomics and lipidomics, 100 flies (50 males + 50 females) were used for each replicate and three replicates were performed. Replicates were not excluded from analyses.
Randomization	Allocation was not random, in most experiments, test sample were compared to control samples.
Blinding	The investigators were not blinded during data collection as the biological groups are well defined and handled in parallel. Instead of blinding, we have relied upon biological replicates and verification of key data by independent techniques where possible.

Reporting for specific materials, systems and methods

We require information from authors about some types of materials, experimental systems and methods used in many studies. Here, indicate whether each material, system or method listed is relevant to your study. If you are not sure if a list item applies to your research, read the appropriate section before selecting a response.

Materials & experimental systems

n/a	Involved in the study
	☒ Antibodies
	☒ Eukaryotic cell lines
☒	Palaeontology and archaeology
	☒ Animals and other organisms
☒	Clinical data
☒	Dual use research of concern

Methods

n/a	Involved in the study
☒	ChIP-seq
☒	Flow cytometry
☒	MRI-based neuroimaging

Antibodies

Antibodies used	Immunohistochemistry:<br>Primary antibodies<br>Mouse anti GFP B-2 1:100 Santacruz Biotech sc9996; rabbit anti GFPrabbit 1:100 Chromotek pabg1; anti V5 1:100 EMD Millipore AB3792; rabbit anti HA C29F4 1:500 Cell Signaling Technology 3724; mouse anti HA 1:500 6E2 Cell Signaling Technology; rabbit anti-DsRed 1:100 Clontech 632596; mouse anti-Cut 1:10 cell culture supernatant DSHB 2B10.<br>Secondary antibodies<br>Goat anti mouse Alexa Fluor Plus 488nm 1:1000 Invitrogen A32723; goat anti mouse Alexa Fluor Plus 555nm 1:1000 Invitrogen A32727; goat anti rabbit Alexa Fluor Plus 488nm 1:1000 Invitrogen A32731, goat anti rabbit Alexa Fluor Plus 555nm 1:1000 Invitrogen A32732; donkey anti-rabbit 568 nm 1:100 ThermoFisher A10042; donkey anti-mouse 647 nm 1:100 ThermoFisher A31571.<br>Immunoblotting:<br>Primary antibodies<br>Mouse anti-GFP B-2 1:250 Santa Cruz Biotechnology sc9996; rabbit anti GFPrabbit 1:1000 Chromotek pabg1; mouse anti-V5 1:500 Invitrogen 46-1157; rabbit anti-HA C29F4 1:1000 Cell Signaling 3724; mouse anti actin 1:1000 DSHB JLA20; mouse anti actin 8H10D10 1: 1000 Cell Signaling 3700..<br>Secondary antibodies<br>Goat anti rabbit HRP or goat anti mouse HRP 1:3000 or 1:5000 Jackson ImmunoResearch.
Validation	The antibodies are widely used by the scientific community and validation has been performed by the manufacturers as well as in previous publications.

Eukaryotic cell lines

Policy information about cell lines and Sex and Gender in Research

Cell line source(s)	Schneider's Drosophila Line 2 (D.Mel. (2), SL2) was obtained from ATCC, CRL-1963.
Authentication	Cell line was not authenticated by us. This cell line is widely used by the Drosophila community.
Mycoplasma contamination	Cell line was not tested for Mycoplasma contamination by us.
Commonly misidentified lines (See ICLAC register)	n/a

Animals and other research organisms

Policy information about studies involving animals; ARRIVE guidelines recommended for reporting animal research, and Sex and Gender in Research

Laboratory animals	5-7 day old Drosophila melanogaster flies were used unless otherwise stated in the study. Tubulin Gal4 (5138), fat body Gal4 (6982), tubulin Gal80ts (7018), LTP RNAi (51937), mCherry NLS (38424), Mex 1 Gal4 (91368), BBB Gal4 (50472), deficiency (7879), nos Cas9 (54591), UAS NaChBac (9469), CaLexA (66542), ilp2,3 mutants (30888), and ilp2,3,5 mutants (30889) were obtained from Bloomington Stock Center. 8093 RNAi (19561), UAS LRP1 RNAi (8397) and UAS Megalin RNAi (36389) were obtained from Vienna Drosophila Resource Center. vaha mutant, UAS Vaha GFP, UAS VahaΔ30 GFP, Vaha V5, VahaΔ30 V5, Vaha Gal4, and Vaha active site mutant flies were generated in this study as described in Methods.
Wild animals	Wild animals were not used in this study.
Reporting on sex	Experiments were performed both in male and female flies.
Field-collected samples	Field-collected samples were not used in this study.
Ethics oversight	Ethical approval was not required for Drosophila studies.

Note that full information on the approval of the study protocol must also be provided in the manuscript.
